# Highly diverged novel subunit composition of apicomplexan F-type ATP synthase identified from *Toxoplasma gondii*

**DOI:** 10.1371/journal.pbio.2006128

**Published:** 2018-07-13

**Authors:** Rahul Salunke, Tobias Mourier, Manidipa Banerjee, Arnab Pain, Dhanasekaran Shanmugam

**Affiliations:** 1 Biochemical Sciences Division, CSIR-National Chemical Laboratory, Pune, Maharashtra, India; 2 Pathogen Genomics Laboratory, BESE Division, King Abdullah University of Science and Technology (KAUST), Thuwal, Saudi Arabia; 3 Kusuma School of Biological Sciences, Indian Institute of Technology-Delhi, Hauz Khas, New Delhi, India; University of Pennsylvania School of Veterinary Medicine, United States of America

## Abstract

The mitochondrial F-type ATP synthase, a multisubunit nanomotor, is critical for maintaining cellular ATP levels. In *T*. *gondii* and other apicomplexan parasites, many subunit components necessary for proper assembly and functioning of this enzyme appear to be missing. Here, we report the identification of 20 novel subunits of *T*. *gondii* F-type ATP synthase from mass spectrometry analysis of partially purified monomeric (approximately 600 kDa) and dimeric (>1 MDa) forms of the enzyme. Despite extreme sequence diversification, key F_O_ subunits a, b, and d can be identified from conserved structural features. Orthologs for these proteins are restricted to apicomplexan, chromerid, and dinoflagellate species. Interestingly, their absence in ciliates indicates a major diversion, with respect to subunit composition of this enzyme, within the alveolate clade. Discovery of these highly diversified novel components of the apicomplexan F-type ATP synthase complex could facilitate the development of novel antiparasitic agents. Structural and functional characterization of this unusual enzyme complex will advance our fundamental understanding of energy metabolism in apicomplexan species.

## Author summary

The mitochondrial F-type ATP synthase, which is a major source of ATP in eukaryotic cells, is a unique nanomotor. The enzyme consists of two subcomplexes called the F_1_ and F_O_ sectors. The F_1_ sector is the site of ATP synthesis, while the F_O_ sector couples proton translocation to the rotary motion of the enzyme. F_O_ sector subunits also form the peripheral stalk (stator), which holds the nonrotating parts of the enzyme together. F_1_ sector subunits are highly conserved among eukaryotes, while most F_O_ sector subunits appear to be diverse and remain unknown in many species, including in the important human pathogen *Toxoplasma gondii*. In this study, we have partially purified the *T*. *gondii* F-type ATP synthase and analyzed its proteome, using mass spectrometry. This resulted in the identification of 20 novel subunits of the enzyme, including extremely diversified key F_O_ sector subunits. Many of these novel *T*. *gondii* proteins are conserved in related apicomplexan parasites, such as the malaria parasite, and thus might be good drug targets. Conservation of many of these proteins in related free-living alveolate species provides insights on their evolutionary history and can potentially facilitate further biochemical and structural studies on this unusual enzyme.

## Introduction

The F-type ATP synthase is a ubiquitous nanomotor present on the inner membrane of mitochondria, chloroplast, and bacterial plasma membrane that catalyzes ATP formation from ADP and Pi [1,2]. In mitochondria, the energy required to drive the nanomotor is harnessed from the proton motive force and associated membrane potential, which are generated by the mitochondrial respiratory complexes [3,4]. The mitochondrial F-type ATP synthase complex consists of two subcomplexes called the F_1_ and F_O_ sectors. The hydrophilic globular F_1_ sector includes three each of α and β subunits and a central stalk containing one each of γ, δ, and ε subunits. The site of ATP formation resides in the catalytic center located in the β subunit [1,2,4,5]. Unlike the F_1_ sector subunit components, which are well conserved across various eukaryotic phyla, many F_O_ sector subunit components are highly diverse [6–9] and are not readily identified based on sequence similarity. The complete set of F_O_ sector subunits have been mapped only in a few species [10,11]. For example, in the yeast *Saccharomyces cerevisiae*, the F_O_ sector is composed of the hydrophobic membrane–spanning oligomeric subunit c (10–12 monomers), along with subunits a, b, d, f, 8, h, and OSCP (oligomycin sensitivity–conferring protein) [10]. In addition, other accessory subunits such as e, g, i, j, and k are also associated with the enzyme. Orthologs of all yeast F_O_ sector subunits, except subunits j and k, are present in the mammalian counterpart (bovine enzyme), which additionally has subunits AGP and MLQ [11].

Numerous biochemical and structural studies have addressed the assembly and interaction between the various subunit components of F-type ATP synthases [1,12]. These studies have provided in-depth insights on the functional coupling of the proton motive force to ATP synthesis. The F_O_ sector subunits c and a interact to form the proton channel; and proton translocation, which occurs along the interface of these two subunits, is coupled to the rotary motion of the c-ring and central stalk of the enzyme [13–16]. The peripheral stalk structure of the enzyme—comprised of F_O_ sector subunits b, d, h (f6 in bovine), and OSCP—has the critical role of holding the α_3_β_3_ catalytic domain in place during the clockwise rotation of the central stalk. These events elicit conformational changes in the α_3_β_3_ catalytic domain, which then results in ATP synthesis [2,4,17,18]. F_O_ sector subunits e and g are known to be involved in dimer assembly of the enzyme complex in yeast [19,20], which appears to facilitate cristae formation by the mitochondrial inner membrane [21–24].

However, despite the importance of the various F_O_ sector subunits, only subunit c and OSCP can be detected from sequence conservation in the vast majority of unicellular eukaryotes, including in the alveolate infrakingdom, which includes the phylum Apicomplexa [25–27]. It is unlikely that the missing subunits are totally absent in these species. Plausible scenarios are that they have either diverged in sequence beyond recognition or that a completely new set of proteins have substituted for the missing subunits in these species. This is indeed the case in organisms such as *Euglena gracilis* [9], *Trypanosoma brucei* [7], *Tetrahymena thermophila* [8], and Chlorophyceae algae [6]. Hence, our interest was to characterize the complete subunit composition of the F-type ATP synthase from the apicomplexan parasite *T*. *gondii*.

*T*. *gondii* is an obligate intracellular protozoan parasite responsible for toxoplasmosis in humans and animals [28]. The parasite completes its life cycle in two host species; the definitive hosts are naïve feline species that support the sexual development of the parasite, and all warm-blooded animals are intermediate hosts and support its asexual development [29]. During asexual development, the parasites can reversibly differentiate between fast-growing virulent tachyzoites and slow-growing latent bradyzoites [30]. By virtue of their fast-growing nature, tachyzoites are metabolically more active, and glycolysis is the primary source for carbon and energy for optimal growth of this parasite [31–33]. However, recent findings have revealed that glucose is not an essential nutrient [34], and parasites survive in the presence of glutamine as an alternative nutrient source [35,36]. In the absence of glucose oxidation via glycolysis, oxidative phosphorylation is the only source for bulk cellular ATP. Evidence for an active oxidative phosphorylation in *T*. *gondii* exists [37–39], and treatment with atovaquone, a potent inhibitor of mitochondrial electron transport chain (mtETC), results in inhibition of ATP synthesis and parasite growth (*EC*_50_ < 50 nM) [40].

Very little is known about the structure and function of the F-type ATP synthase from *T*. *gondii* and other apicomplexan parasites. Comparative genomics has revealed that, while the five canonical F_1_ sector subunits—α, β, γ, δ, and ε—are readily identified in all apicomplexan parasites based on amino acid sequence conservation, only two F_O_ sector subunits—c and OSCP—can be identified by sequence [25–27]. Characterization of F-type ATP synthase from solubilized mitochondria lysates of the human malaria parasite *Plasmodium falciparum* revealed that the enzyme assembles into monomer and dimer forms [26]. This suggests that a full complement of subunits, typical for the eukaryotic enzyme, is present in *P*. *falciparum* and likely in other apicomplexan parasites as well.

Here, we have investigated the subunit composition of the enzyme from *T*. *gondii*. We were able to identify and partially purify the F-type ATP synthase enzyme complex from solubilized mitochondrial preparation of *T*. *gondii* using blue native PAGE (BNP) separation, by immunoprecipitation (IP) and by chromatographic enrichment. Liquid chromatography–mass spectrometry (LC-MS) analysis of the enzyme preparations revealed the identity of the proteins associated with the *T*. *gondii* F-type ATP synthase. We have identified 20 novel proteins (of unknown function) as being bona fide subunit constituents of *T*. *gondii* F-type ATP synthase, based on consensus from multiple and independent experiments. Phyletic profiling revealed that orthologs for many of these proteins are present in most apicomplexan species, except in those *Cryptosporidium* spp. that possess a degenerate mitochondrion devoid of all proteins involved in oxidative phosphorylation [41]. Further, the phyletic profiles of these novel proteins reveal their origin to be ancient, probably even ancestral to alveolate radiation. The identification of these novel protein components of the apicomplexan F-type ATP synthase will facilitate further structural, functional, and inhibitor discovery studies on this important enzyme complex.

## Results

### Identification of F-type ATP synthase subunits from *T*. *gondii* proteome

**Fig 1:**
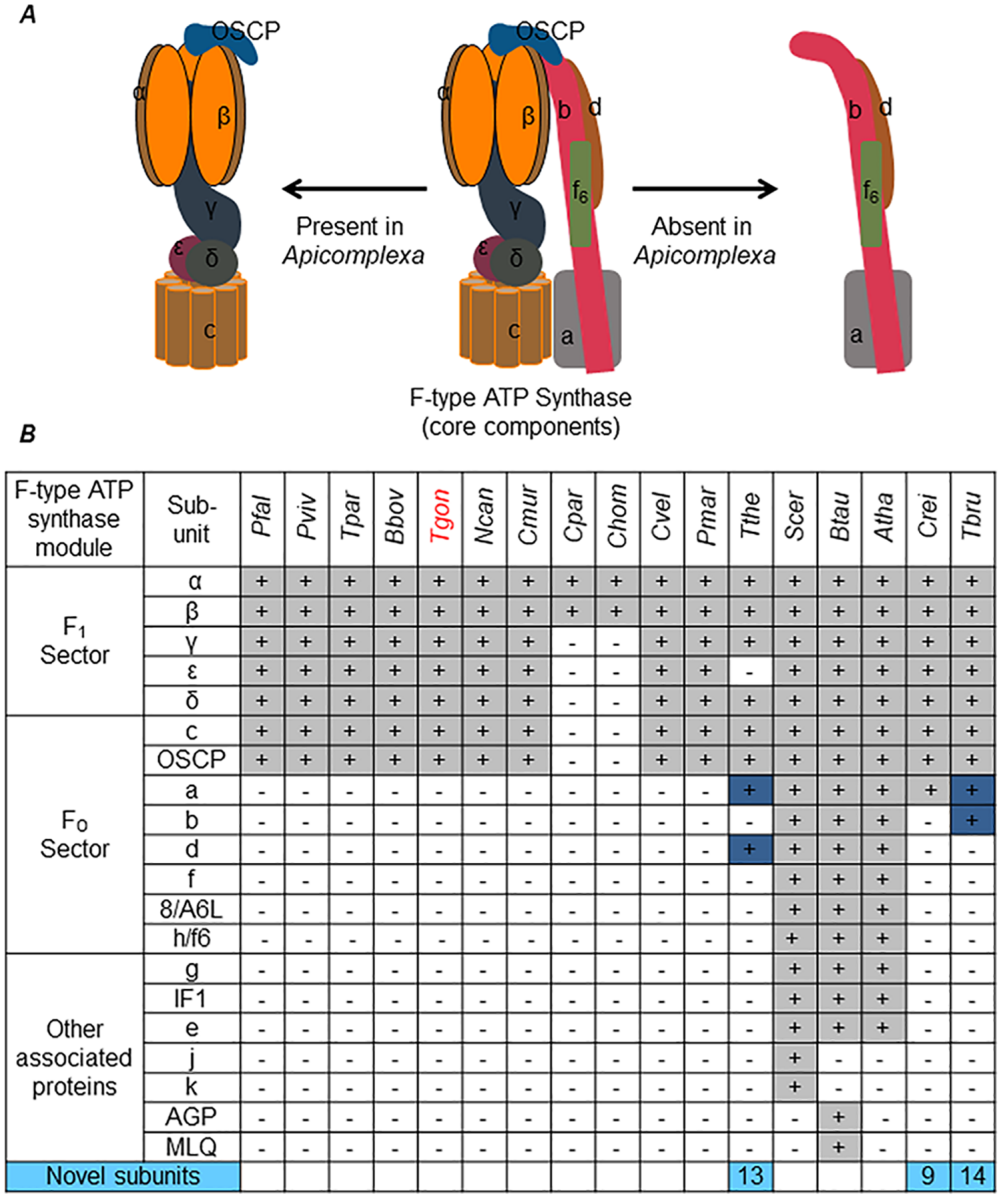
Detail of F-type ATP synthase subunits missing in *T*. *gondii* and other apicomplexan parasites. (A) Schematic representation of core subunit composition of F-type ATP synthase. The different subunits are color coded and annotated. Middle, the complete set of subunits of the core enzyme; left and right, subunits identified and not identified by in silico methods in apicomplexan genome. (B) Table showing the presence/absence of orthologs of yeast and bovine F-type ATP synthase in *Apicomplexa* and other alveolate species. +/−, presence/absence of ortholog; blue, diverged (no clear orthologs) functional equivalent; light blue, experimentally identified species-specific novel subunits (numeric values indicate the total number of such novel subunits). Species names: *Pfal*, *P*. *falciparum*; *Pviv*, *Plasmodium vivax*; *Tpar*, *Theileria parva*; *Bbov*, *Babesia bovis*; *Tgon*, *T*. *gondii*; *Ncan*, *Neospora caninum*; *Cmur*, *Cryptosporidium muris*; *Cpar*, *Cryptosporidium parvum*; *Chom*, *Cryptosporidium hominis*; *Cvel*, *Chromera velia*; *Pmar*, *Perkinsus marinus*; *Tthe*, *T*. *thermophila*; *Scer*, *Saccharomyces cerevisiae*; *Btau*, *Bos taurus*; *Atha*, *Arabidopsis thaliana*; *Crei*, *Chlamydomonas reinhardtii*; *Tbru*, *T*. *brucei*. IF1, inhibitory factor 1; OSCP, oligomycin sensitivity–conferring protein.

Comprehensive in silico analysis on the presence and absence of key enzymes involved in mitochondrial metabolism in apicomplexan species has been previously carried out [25,27,42]. These studies have highlighted the missing subunits of the F-type ATP synthase enzyme from these organisms. We have further confirmed these findings from our efforts to identify *T*. *gondii* orthologs for yeast and bovine F-type ATP synthase subunits. Orthologs for F_1_ sector subunits α (TGME49_204400), β(TGME49_261950), δ(TGME49_226000), ε(TGME49_314820), and γ(TGME49_231910) and F_O_ sector subunits c (TGME49_249720) and OSCP (TGME49_284540) were readily identified from *T*. *gondii*. This suggested that all the subunits necessary for assembling the catalytic core (α3β3), central stalk (δ, ε, and γ), and the subunit c oligomer portions of the F-type ATP synthase (Fig 1A) are encoded by *T*. *gondii*. However, as reported previously [25,27], we were unable to identify the orthologs for critical F_O_ sector subunits involved in proton translocation and formation of the peripheral stalk structure of the enzyme in *T*. *gondii* through routine bioinformatic analysis. This appears to be the case in all apicomplexan species and in other taxons within the alveolate infrakingdom, such as the dinoflagellates, ciliates, and chromerids (Fig 1B). It is likely that the missing F_O_ subunits are either highly divergent or completely novel. This situation is not exclusive to alveolates, since many other unicellular eukaryotes also appear not to possess the corresponding orthologs for many of the yeast or bovine F_O_ subunits. In fact, novel F-type ATP synthase–associated proteins (ASAPs) have been previously identified from *Tetrahymena* [8], *Chlamydomonas* [6], and *Trypanosoma* [7]. Therefore, we attempted to purify the F-type ATP synthase enzyme from isolated *T*. *gondii* mitochondria in native form and identify its protein composition by mass spectrometry.

### Generating transgenic parasites expressing *T*. *gondii* F-type ATP synthase subunits β (*Tg*ATPβ) and OSCP (*Tg*ATPOSCP) tagged with yellow fluorescent protein plus hemagglutinin (YFP-HA)

**Fig 2:**
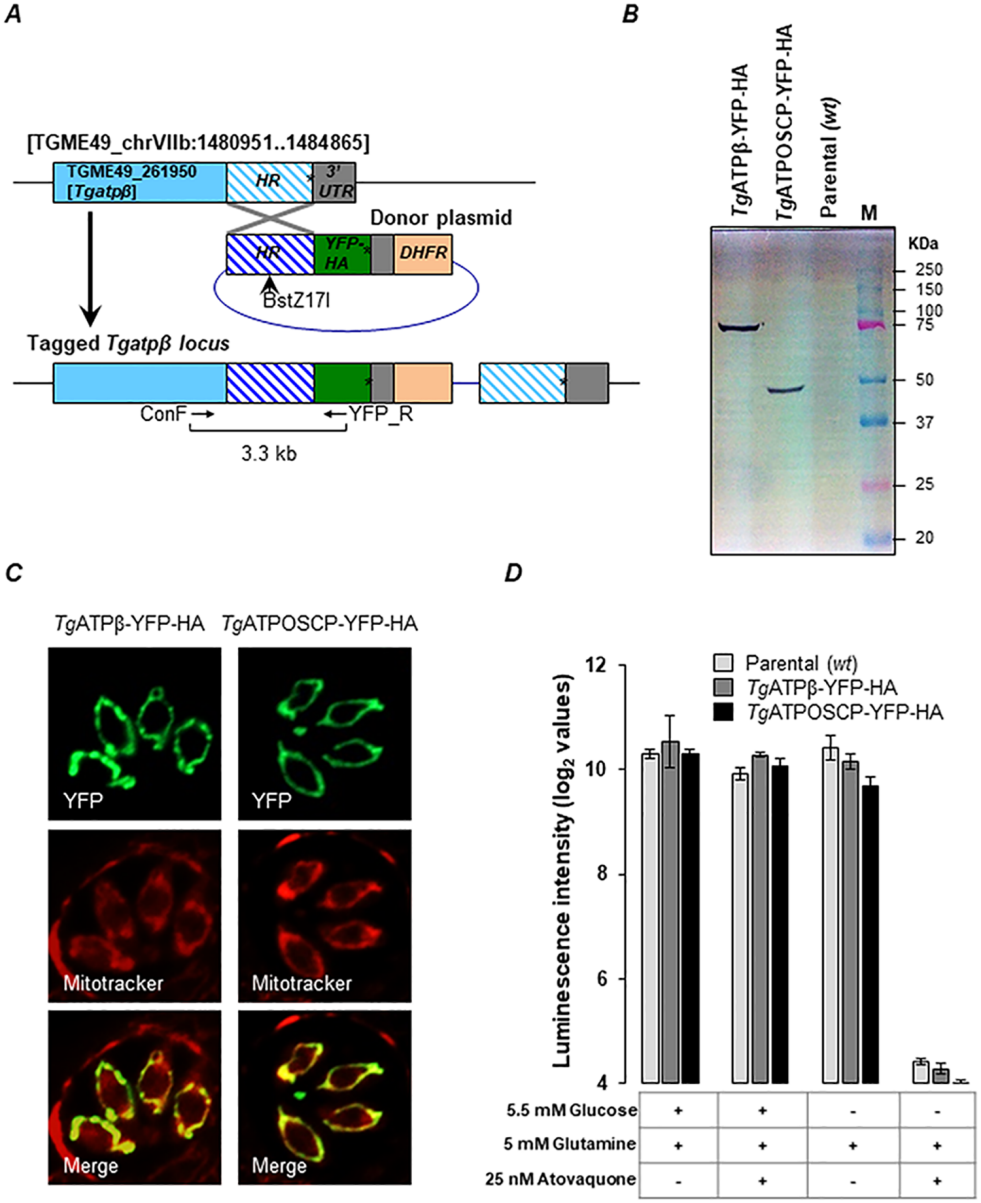
Endogenous tagging of *Tgatpβ* and *Tgatposcp* genes with YFP-HA tags. (A) Schematic representation of the *Tgatpβ*(TGME49_261950) gene locus showing coding region in light blue and 3′ UTR in gray. The hatched region shows the HR, which was cloned into a donor plasmid (dark blue) upstream of an in-frame coding region for YFP-HA tag. The donor plasmid also includes the DHFR cassette, which confers pyrimethamine resistance. Asterisk denotes the position of stop codon. The donor plasmid was linearized with the restriction enzyme BstZ17I to facilitate single-crossover homologous recombination, and the resulting modification of the gene locus was confirmed by PCR using the primers ConF and YFP_R. A similar strategy was used to tag the *Tgatposcp* gene locus. (B) and (C) Confirming the expression of the *Tg*ATPβ-YFP-HA and *Tg*ATPOSCP-YFP-HA proteins by SDS-PAGE western blotting (B) and microscopy (C) on respective clonal transgenic lines. Gel lanes in (B): 1, Cell lysate from *Tg*ATPβ-YFP-HA transgenic parasites; 2, Cell lysate from *Tg*ATPOSCP-YFP-HA transgenic parasites; 3, Cell lysate from parental parasites; M, Molecular weight size markers. Mitotracker was used to visualize the mitochondrion in (C). (D) Functional validation of mitochondrial ATP synthesis in *wt* parental strain, and *Tg*ATPβ-YFP-HA- and *Tg*ATPOSCP-YFP-HA-expressing transgenic parasites. All three strains exhibited similar response to atovaquone treatment in the presence and absence of glucose, thereby confirming that the YFP-HA tag had no effect on mitochondrial ATP synthesis. The raw luminescence reading from this experiment and its analysis are provided in S1 Data file. DHFR, dihydrofolate reductase; HR, homology region; *Tg*ATPβ, *T*. *gondii* ATP synthase β subunit; *Tg*ATPOSCP, *T*. *gondii* ATP synthase OSCP subunit; wt, wild type; YFP-HA, yellow fluorescent protein plus hemagglutinin.

To facilitate the purification of the *T*. *gondii* F-type ATP synthase enzyme in native form, we engineered in-frame genomic YFP-HA tags in the 3′ end of the respective genes encoding the F_1_ β and F_O_ OSCP subunits (Fig 2A). The modified genes continued to be expressed under the control of their endogenous promoters. The correct insertion of the tags was confirmed by genomic PCRs (S1 Fig) from clonal isolates of the respective transgenic parasites. Expression of the desired YFP-HA tagged proteins and their mitochondrial localization were confirmed by western blotting (Fig 2B) and microscopy (Fig 2C), respectively. We then compared the ability of the parental and transgenic strains of the parasites to maintain total cellular ATP levels in the presence/absence of glucose. Although *T*. *gondii* tachyzoites are known to prefer glucose as the primary nutrient source, in the absence of glucose, they are known to switch to glutaminolysis for carbon and energy supply. In the latter case, ATP is obtained by the parasites via oxidative phosphorylation, and this can be inhibited using atovaquone [25,40,43]. Like the parental parasites, transgenic parasites expressing YFP-HA-tagged F_1_ β and F_O_ OSCP subunits were capable of maintaining cellular ATP levels via oxidative phosphorylation in the absence of glucose, which was inhibited by atovaquone (Fig 2D). These results confirm that the modification of either F_1_ β or F_O_ OSCP proteins with YFP-HA tag had no detrimental effect on the function of the enzyme, implicating that the structure of the tagged enzyme remained intact.

### Identification of monomer and dimer forms of *T*. *gondii* F-type ATP synthase by BNP analysis

**Fig 3:**
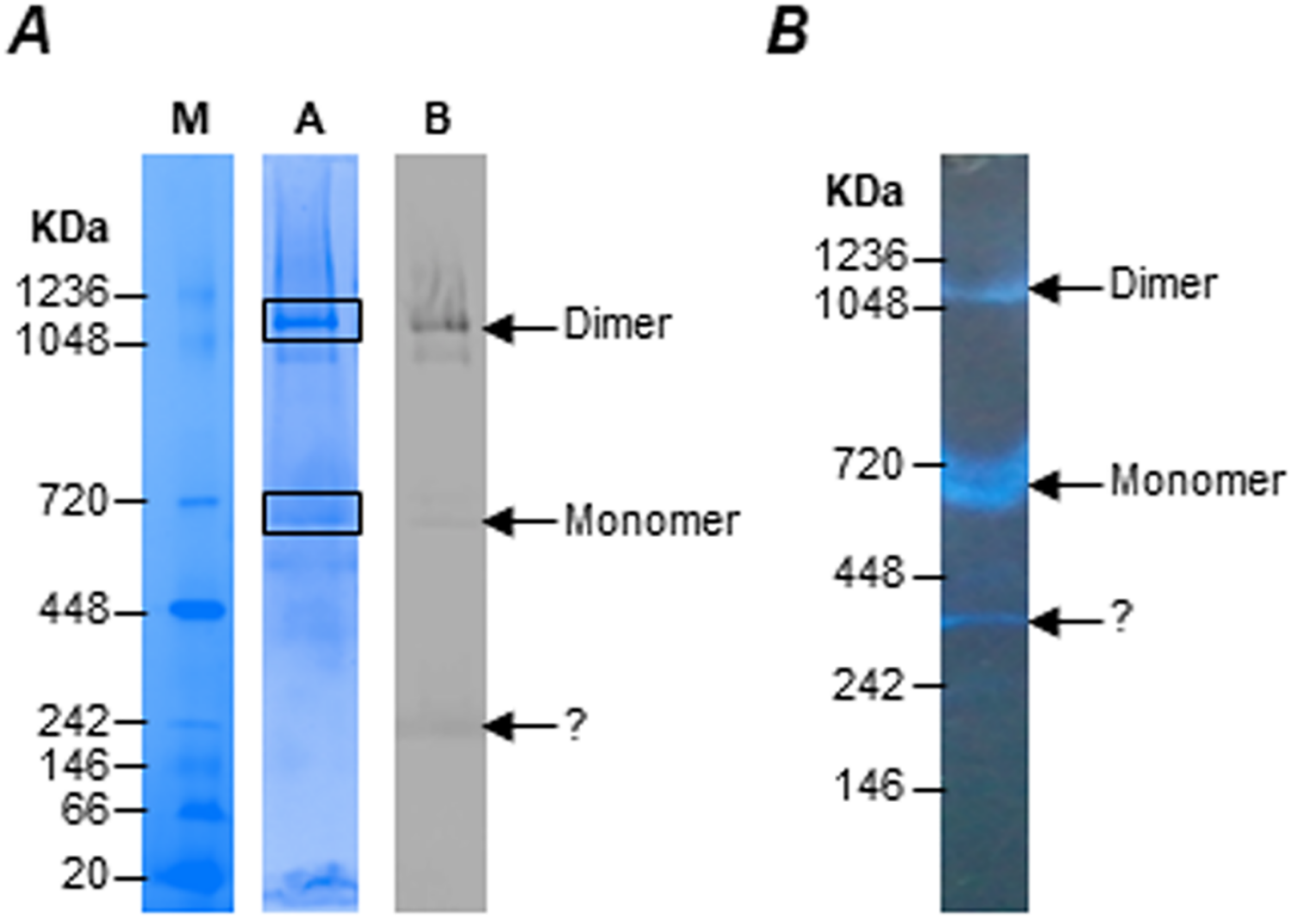
Identifying the dimer and monomer forms of *T*. *gondii* F-type ATP synthase by BNP analysis. (A) Mitochondria lysates were prepared from tachyzoites stage transgenic parasites expressing *Tg*ATPβ-YFP-HA protein were separated by BNP. Lane M, native molecular weight markers; lane A, Coomassie blue staining of BNP gel; lane B, western blotting of BNP gel using α-HA antibodies. The dimer and monomer forms are indicated by arrows. The boxed regions in lane A correspond to the excised gel pieces, which were processed for LC-MS/MS analysis. (B) In-gel ATPase activity assays following BNP separation confirms that the dimer and monomer forms of F-type ATP synthase are functionally intact. BNP, blue native PAGE; HA, hemagglutinin; LC-MS/MS, liquid chromatography–tandem mass spectrometry; *Tg*ATP-β, *T*. *gondii* ATP synthase β subunit; *Tg*ATP-OSCP, *T*. *gondii* ATP synthase OSCP subunit; YFP-HA, yellow fluorescent protein plus hemagglutinin.

The fully assembled F-type ATP synthase is known to exist as dimers on the inner mitochondrial membrane, and this dimerization is known to influence the characteristic cristae formation by the membrane [21,24]. In the case of the human malaria parasite *P*. *falciparum* F-type ATP synthase, dimer formation has been observed previously [26]. To find out whether the *T*. *gondii* enzyme can assemble into dimers, we carried out BNP analysis of detergent-solubilized mitochondrial preparations. After resolving the samples on a 3%–12% gradient BNP gel capable of resolving a molecular weight range between 20 kDa to approximately 1,200 kDa (Fig 3A lane M), Coomassie staining revealed prominent bands at the sizes corresponding to dimeric and monomeric forms of the enzyme (Fig 3A lane A). This was further confirmed by western blotting after BNP separation using α-HA antibodies (Fig 3A lane B), which indicated that the dimer form of the enzyme is more abundant than the monomer form.

Next, we carried out in-gel activity assays to detect the ATPase activity associated with the isolated enzyme. Results from these assays confirmed that the ATPase activity was intact, for both dimer and monomer forms of the enzyme, after BNP separation (Fig 3B). We found that, although the dimer form of the enzyme was more abundant, its ATPase activity was less than that of the monomer form. This is supported by the fact that F_1_ ATPase activity is inhibited in vivo to minimize the risk of ATP hydrolysis and favor ATP synthesis [44]. Since the in-gel activity assay is an ATP hydrolysis assay, the dimer has less of this activity. Based on BNP mobility, we deduce the size of the dimer form of the enzyme to be 1–1.2 MDa and that of the monomer form to be approximately 600 kDa. This is in agreement with what has been reported previously for *P*. *falciparum* [26], *S*. *cerevisiae* [19], and bovine [45] enzymes and suggests that a full complement of F_O_ subunits is present in *T*. *gondii* F-type ATP synthase. Therefore, we excised the regions in the BNP corresponding to the dimer and monomer forms of the enzyme and proceeded to identify the proteins in the gel band by LC-MS analysis (Fig 3A).

### Identification of *T*. *gondii* F-type ASAPs by liquid chromatography–tandem mass spectrometry (LC-MS/MS) proteomics

A standard protocol (see Methods section) was followed for in-gel LC-MS/MS analysis of the gel bands corresponding to the dimer and monomer forms of the enzyme identified by BNP. This was done on samples prepared from both *Tg*ATPβ–YFP-HA and *Tg*ATPOSCP–YFP-HA expressing transgenic parasite lines. Since the dimer form of the enzyme was more abundant than the monomer form after BNP separation, we had better success with LC-MS/MS analysis of the gel band corresponding to the dimer form of the enzyme. A total of 96 proteins were identified with high confidence from consensus data obtained from multiple in-gel LC-MS/MS analysis (S1 Table). Proteins corresponding to all F_1_ subunits and F_O_ OSCP were detected, while F_O_ subunit c was not detected in any of the experiments. In addition, many proteins of unknown function were also detected. However, due to comigration of several nonspecific proteins, such as myosin, in the area corresponding to the gel band processed for LC-MS/MS analysis, it is likely that some of these proteins of unknown function are not bona fide subunits of the F-type ATP synthase. A full list of the proteins identified from these samples, along with the details of the peptides detected, is given in S1 Table.

**Fig 4:**
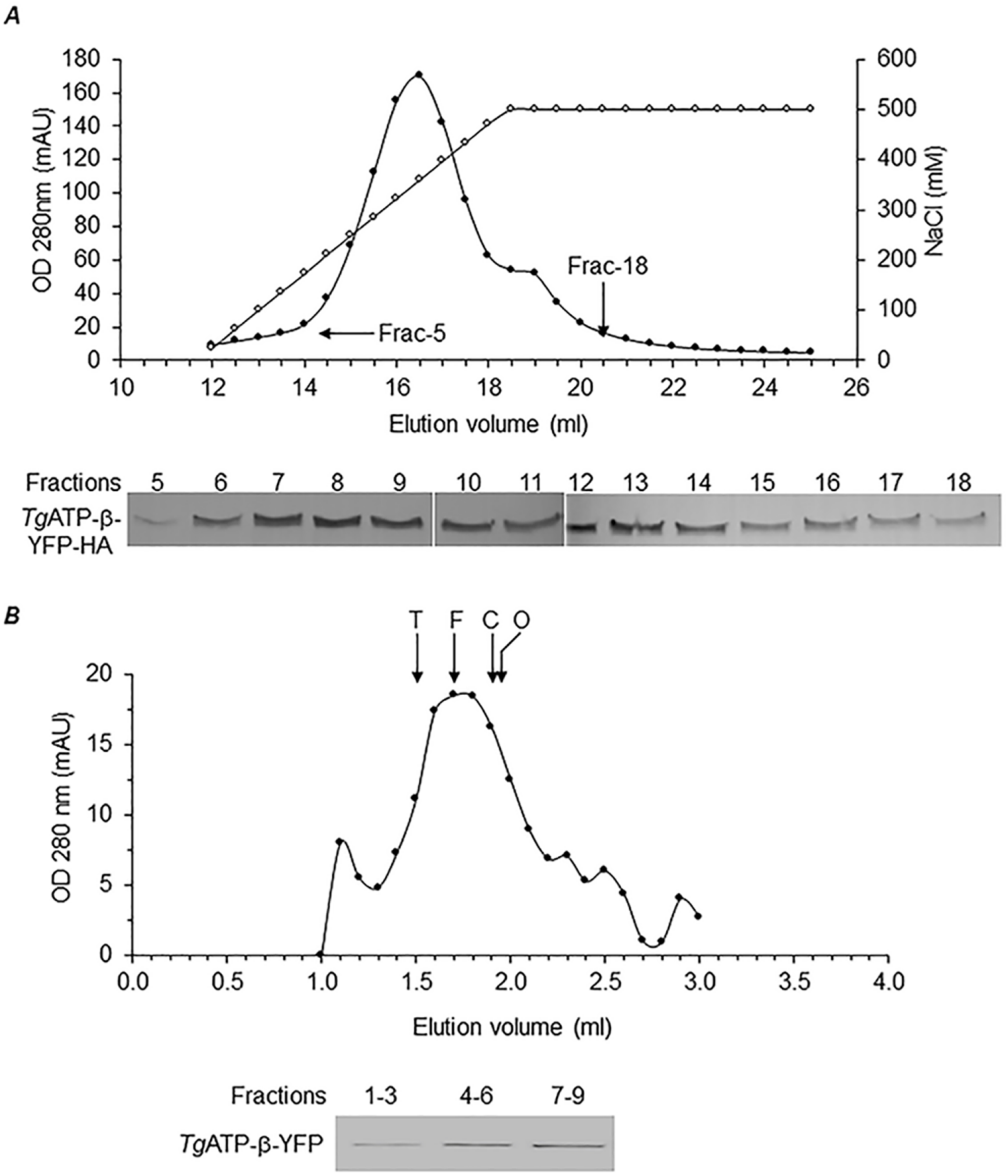
Partial purification of dimer and monomer forms of *T*. *gondii* F-type ATP synthase by chromatography. (A) Ion exchange (DEAE sepharose) separation of mitochondrial lysates prepared from *Tg*ATPβ-YFP-HA expressing transgenic parasites. Absorbance at 280 nm (filled circles) and NaCl concentration (open circles) are plotted for each fraction. Fractions 5 and 18 are marked with arrows. Bottom panel shows SDS-PAGE western blotting for fractions 5–18 to find out the elution profile of *Tg*ATPβ-YFP-HA. (B) Size exclusion profile of the pooled fractions from ion exchange chromatography. The absorbance at 280 nm is plotted for each fraction. The size exclusion column was calibrated with the following native markers: thyroglobulin (labeled “T,” 660 kDa), ferritin (labeled “F,” 440 kDa), conalbumin (labeled “C,” 75 kDa), Ovalbumin (labeled “O,” 45 kDa). Peak elution volume for each marker is indicated by arrow. Fractions 1–3, 4–6, and 7–9 were pooled, concentrated, and subject to SDS-PAGE western blotting to detect *Tg*ATPβ-YFP-HA, as shown in bottom panel. DEAE, diethylaminoethanol; OD, optical density; *Tg*ATPβ, *T*. *gondii* ATP synthase β subunit; YFP-HA, yellow fluorescent protein plus hemagglutinin.

**Fig 5:**
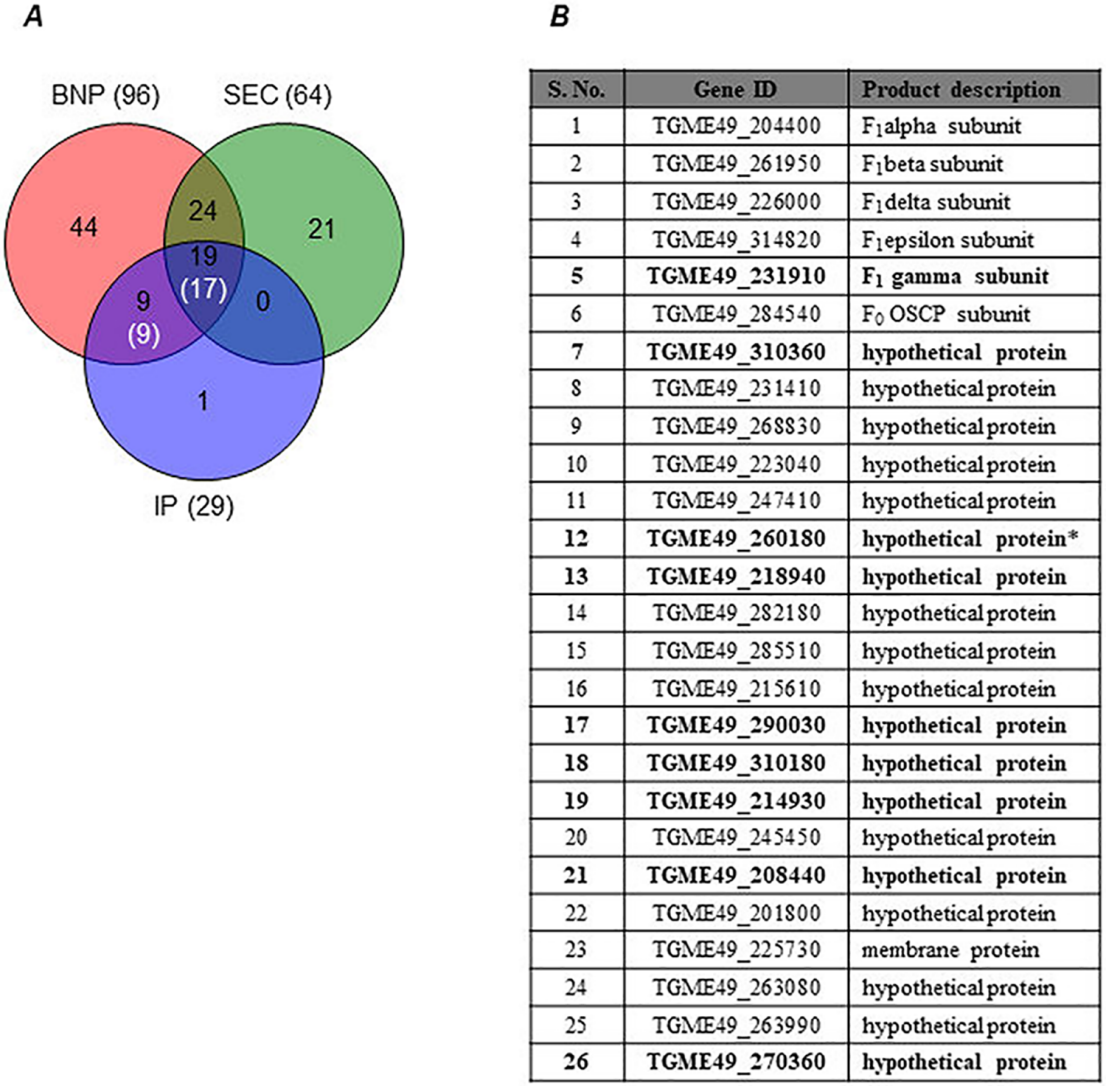
Identification of novel *T*. *gondii* F-type ATP synthase subunits from LC-MS/MS analysis. (A) The Venn diagram shows shared identification of proteins following BNP, SEC, and IP sample preparation. Total number of proteins identified in each technique is given within brackets outside the Venn diagram. The numbers shown in white font and within brackets are the final set of proteins assigned as subunit components of *T*. *gondii* F-type ATP synthase. (B) List of all genes identified in this work by mass spectrometry analysis. Gene ID and product description details are from Toxodb.org (release 36). The entries shown in bold indicate that the protein was detected with high confidence from only BNP and IP samples. Asterisk indicates that the corresponding peptides were detected in only one of the replicate runs for SEC sample. BNP data are a combination of experiments done with both *Tg*ATPβ-YFP-HA–and *Tg*ATPOSCP-YFP-HA–expressing transgenic parasites. SEC and IP data are from *Tg*ATPβ-YFP-HA and *Tg*ATPOSCP-YFP-HA expressing transgenic parasites, respectively. BNP, blue native PAGE; IP, immunoprecipitation; LC-MS/MS, liquid chromatography–tandem mass spectrometry; OSCP, oligomycin sensitivity–conferring protein; SEC, size exclusion chromatography; *Tg*ATPβ, *T*. *gondii* ATP synthase β subunit; *Tg*ATPOSCP, *T*. *gondii* ATP synthase OSCP subunit; YFP-HA, yellow fluorescent protein plus hemagglutinin.

In order to specifically identify the protein subunits of F-type ATP synthase, we resorted to partially purifying the enzyme before LC-MS/MS analysis by chromatographic separation (Fig 4A and 4B) and IP (using the α-HA antibody). In contrast to BNP analysis, after size exclusion chromatography, we observed that the dimer form of the enzyme was less abundant than the monomer form (Fig 4B). This is likely due to stability issues with the dimer, which might progressively fall apart as monomers during the process of chromatographic separation. The fractions corresponding to the dimer and monomer forms of the enzyme were pooled and concentrated separately before processing for LC-MS/MS analysis. A total of 64 proteins were detected (Fig 5A) from the fractions corresponding to the monomer form of the enzyme, and we did not get reliable data from the dimer fractions, owing to very low protein concentration.

LC-MS/MS analysis on the enzyme enriched by IP resulted in the identification of a total of 29 proteins, of which 28 were also detected from either BNP or chromatography samples (Fig 5A). Details for the peptides detected from chromatography and IP samples are provided in S1 Table. It is notable that 19 proteins, including most F_1_ subunits and F_O_ OSCP, were detected in samples prepared by all three methods. Out of the 29 proteins, only 3 were considered as nonspecific proteins and not related to F-type ATP synthase. Out of the remaining 26 proteins, 5 were known F_1_ subunits (α, β, δ, ε, γ), 1 was F_O_ OSCP, and the remaining 20 were proteins of unknown function. It should be noted that we were unable to identify F_O_ subunit c in any of our LC-MS/MS analyses, probably owing to its highly hydrophobic nature. Based on the consensus of proteins identified by the three different approaches, the F-type ATP synthase from *T*. *gondii* appears to be comprised of at least 27 protein subunits. We have coined the term “ATP synthase–associated proteins” (or “ASAPs”) to refer to the 20 novel subunit components of the enzyme identified in this study. A complete list of all *T*. *gondii* F-type ATP synthase subunits identified by mass spectrometry, along with their annotation from *ToxoDB* and the experiments from which they were detected, is given in the table in Fig 5B.

### Identification of F_O_ subunits a, b, and d

**Fig 6:**
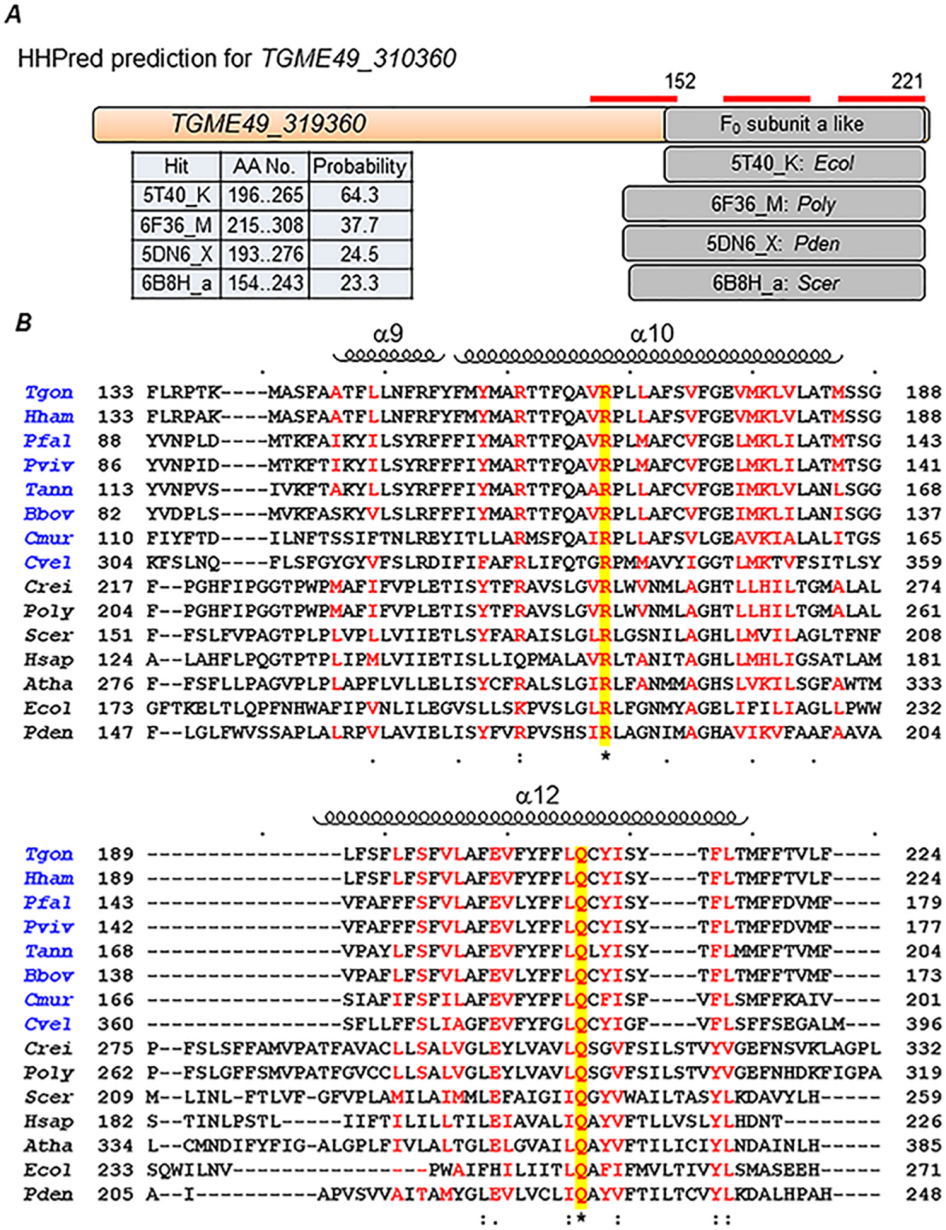
HHPred identification of novel F_O_ subunit a based on conserved structural features. (A) Representation of the pairwise sequence alignments generated by HHPred [46,47] for the putative F_O_ subunit a from *T*. *gondii* and 4 other F_O_ subunit a proteins for which the structure is known. The table provides details of the amino acid length and a probability score for the prediction from the hit alignments. The red lines indicated the 3 transmembrane domains present in the *T*. *gondii* protein. (B) Protein sequence alignments and secondary structure information were made using the Clustal Omega [48] and ESPript [49] software, and only the C-terminal portion of respective proteins is shown. The names of alveolate species are highlighted in blue. For the nonalveolate species included in the alignment, the F_O_ subunit a is either readily identified from sequence (*Scer*, *Hsap*, *Atha*, *Ecol*, and *Pten*) or has been experimentally determined (*Crei* and *Poly*). Positions with similar amino acids are highlighted in red in the alignment. The arginine and glutamine residues, highlighted in yellow, are conserved in all species and important for function. The helices (α9, α10, and α12) shown are from the structure of *Poly* F-type ATP synthase. Species names: *Tgon*, *T*. *gondii*; *Hham*, *Hammondia hammondi*; *Pfal*, *P*. *falciparum*; *Pviv*, *P*. *vivax*; *Tann*, *Theileria annulata*; *Bbov*, *B*. *bovis*; *Cmur*, *C*. *muris*; *Cvel*, *C*. *velia*; *Crei*, *C*. *reinhardtii*; *Poly*, *Polytomella* sp.; *Scer*, *S*. *cerevisiae*; *Hsap*, *Homo sapiens*; *Atha*, *A*. *thaliana*; *Ecol*, *Escherichia coli*; *Pden*, *Paracoccus denitrificans*.

As shown in Fig 1, in addition to subunits c and OSCP, the F_O_ sector consists of at least 6 other subunits in yeast, mammalian, and plant enzymes. Subunit a is essential for proton conductance, which it facilitates along with subunit c [13–15]. Subunit b forms the core of the stator structure, which is essential for holding the catalytic α_3_β_3_ structure in place during the rotary motion of the central stalk [18]. Subunit d is part of the stator structure. We attempted to find out which of the ASAPs correspond to these three subunits in *T*. *gondii* F-type ATP synthase. Since none of the ASAPs had any sequence identity to the yeast F_O_ a, b, and d subunits, we resorted to conserved structure-based identification using pairwise comparison of profile hidden Markov models as implemented in HHPred [46,47]. For this analysis, we used the corresponding tool available from web-based MPI bioinformatics toolkit (toolkit.tuebingen.mpg.de). We analyzed each of the ASAPs using this tool and successfully identified hits for F_O_ subunits a, b, and d based on previously known structures for these proteins from other species. The ASAP TGME49_310360 was identified as F_O_ subunit a (Fig 6A), and we were also able to generate sequence alignments for the C-terminal domain of this protein, which showed the conservation of key amino acid residues arginine (important for proton translocation) and glutamine (yellow highlight in Fig 6B). Similarly, searches with TGME49_231410 and TGME49_268830 came up with hits for F_O_ subunits b and d, respectively (S2 and S3 Figs).

### Phylogenetic analysis reveals conserved F-type ATP synthase subunit composition in 3 major alveolate taxons

**Fig 7:**
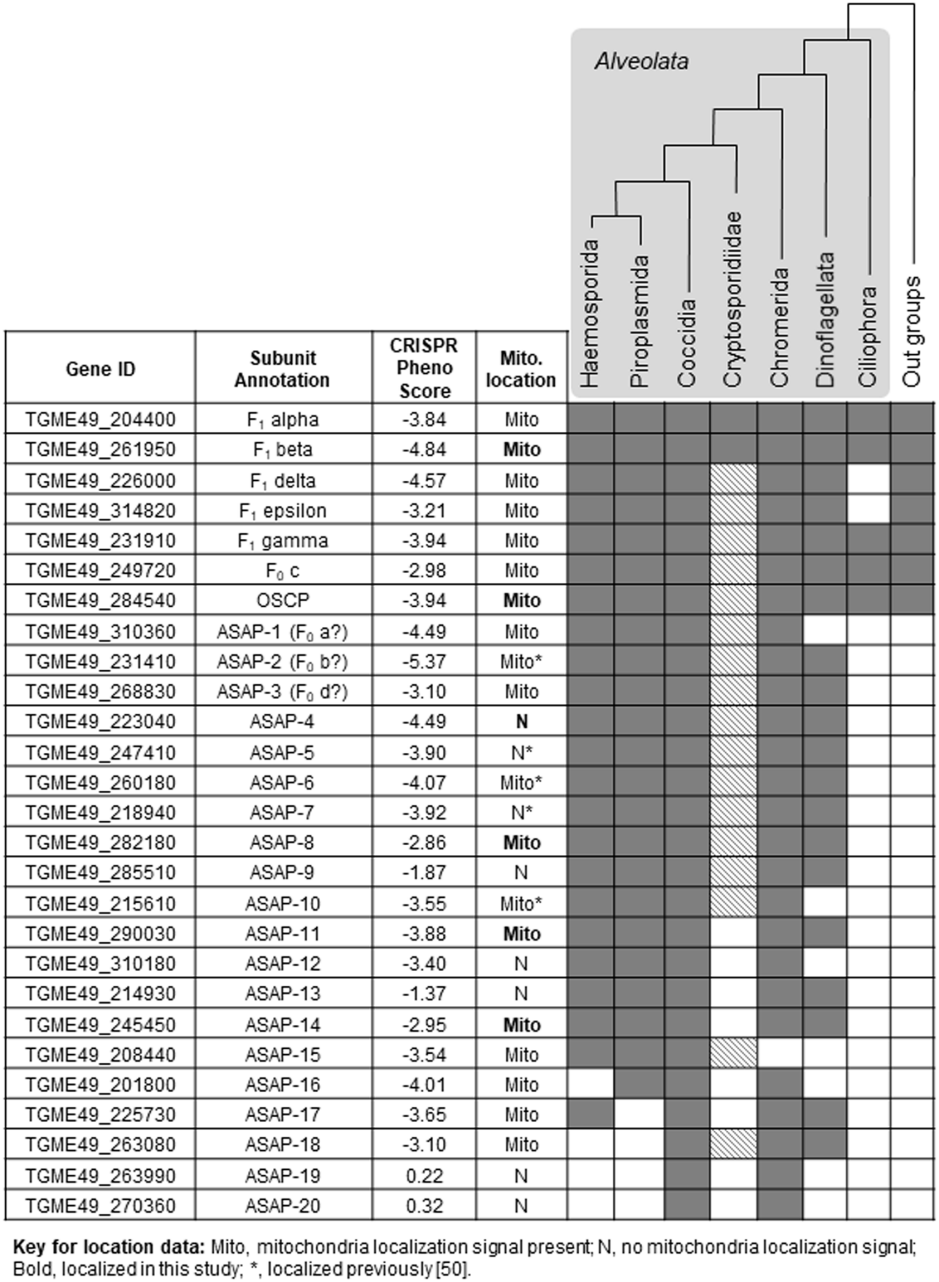
Phylogenetic profile of the alveolate infrakingdom for all *T*. *gondii* F-type ATP synthase subunits. Alveolate clades are highlighted with a gray background, and their expected phylogenetic relationship is indicated by a tree structure above. Gray and white boxes indicate the presence and absence of the corresponding ortholog, respectively. The hatched boxes represent the presence of the ortholog in *C*. *muris* only and absence in *C*. *parvum* and *C*. *hominis*. The table on the left lists the gene ID for all ASAPs, along with their annotation, essentiality phenotypes (phenotype score from CRISPR/Cas9 knockout study [50]), and protein localization. The key for localization annotation is given below the table. ASAP, ATP synthase–associated protein; Cas9, CRISPR-associated 9; CRISPR, clustered regularly interspaced short palindromic repeat; OSCP, oligomycin sensitivity–conferring protein.

From our ortholog identification attempts, and from previous studies [27,42], it was apparent that the F-type ATP synthase subunits missing from *T*. *gondii* were also missing from all other species grouped within the Alveolata infrakingdom. In fact, the novel subunit components identified from *Tetrahymena* (Alveolata; Ciliophora) enzyme were found to be unique to ciliates and not conserved in other alveolate organisms [8]. Therefore, we were interested in finding out whether the novel ASAPs identified in this study are unique to *T*. *gondii* F-type ATP synthase. We were able to identify orthologs for many of the ASAPs in 3 major alveolate taxons—Apicomplexa, Chromerida, and Dinoflagellata (Fig 7). A list of all orthologs identified from selected species belonging to these taxons are given in S2 Table. Out of the 20 ASAPs, 15 were conserved in all apicomplexan clades, except in the case of *Cryptosporidium*, in which only *C*. *muris* contained orthologs for 10 of these proteins. A few ASAPs were unique to the Coccidian clade. More importantly, all ASAPs, except one, were conserved in Chromerida, and at least 9 and 12 ASAPs were also conserved in *Symbiodinium* and *Perkinsus*, respectively.

To obtain further insights on the evolutionary origin of these proteins, we generated neighbor-joining phylogenetic trees for ortholog sequences of all F_1_ and F_O_ subunits from representative species of Haemosporida, Piroplasmida, Coccidia, Cryptosporidiidae, Chromerida, Dinoflagellata, and Ciliophora (Fig 7 and S4 Fig). Stramenopile (*Ectocarpus siliculosis*, *Phaeodactylum tricornutum*, *Pythium ultimum*, *Thalassiosira pseudonana*) and Plantae (*C*. *reinhardtii* and *A*. *thaliana*) species were included as outgroups while constructing the phylogenetic trees for the highly conserved F_1_ subunits. Even though the topology of most trees did not reflect the expected evolutionary relationship between the included species, monophyletic grouping was observed in general at the taxon level, and importantly, this was evident for the conserved F_1_/F_O_ subunits, as well as the novel ASAPs (S4 Fig). Thus, the evolutionary origin of the newly identified highly divergent ASAPs in Apicomplexa, Chromerida, and Dinoflagellata clades appears to be ancient.

### Mitochondrial localization, essentiality, and gene coexpression analysis of ASAPs from *T*. *gondii* and *P*. *falciparum*

**Fig 8:**
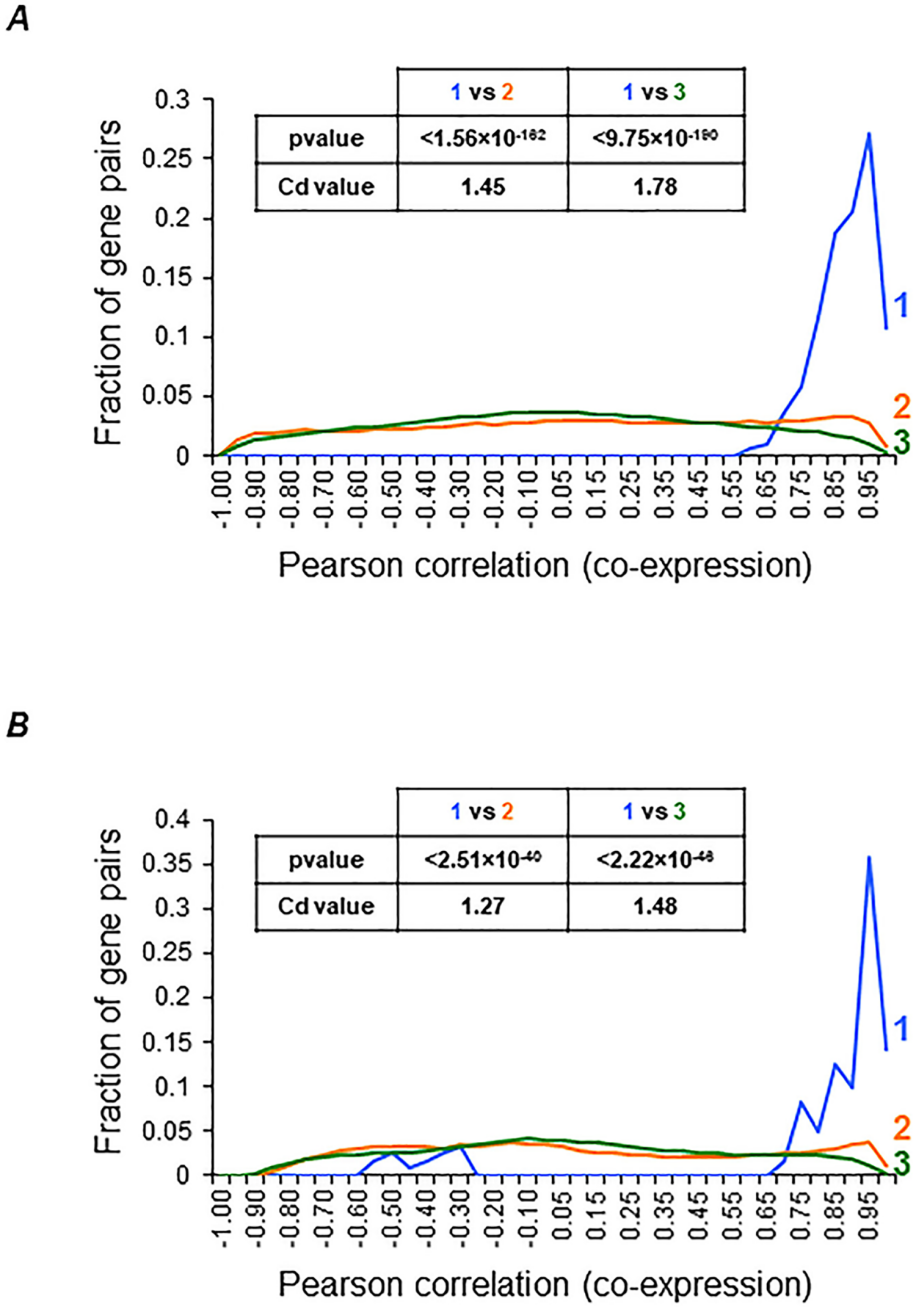
Gene coexpression analysis for *T*. *gondii* and *P*. *falciparum* F-type ATP synthase subunits. The distribution of coexpression values—as measured by the Pearson correlation coefficients—are plotted for the three gene pairs categories, as shown for *T*. *gondii* (A) and *P*. *falciparum* (B). The statistical support from MWU and Cd values are shown in the table within each plot. Blue (1), coexpression correlation between ATP synthase genes; Orange (2), coexpression correlation between ATP synthase genes and non-ATP synthase genes; Green (3), coexpression correlation between non-ATP synthase genes. MWU, Mann-Whitney U *p*-values; Cd, Cohen’s d.

Confirming the mitochondrial localization for the ASAPs is essential for validating their subunit membership in *T*. *gondii* F-type ATP synthase. We first predicted the presence of signal sequence for mitochondrial targeting using the Mitoprot tool [51] in 12 ASAPs (Fig 7). Five ASAPs, including TGME49_231410 (F_O_ subunit b like protein), were previously shown to localize in the mitochondrion [50], and we experimentally confirmed the mitochondrial localization for another four (S5 Fig). Due to the functional importance of the F-type ATP synthase enzyme, it is reasonable to expect that the enzyme would be essential in *T*. *gondii*. This is indeed the case, and all known subunits and ASAPs (except ASAP-19 and 20; Fig 7) of the enzyme were found to be essential in a previous study [50]. In order to obtain further independent evidence for ASAPs as bona fide subunits of F-type ATP synthase, we carried out transcriptome coexpression correlation analysis using publicly available gene expression datasets for *T*. *gondii* and *P*. *falciparum* [52,53]. We assumed that the ASAPs interacting with one other would show significant pairwise correlation profiles in their expression levels. Strikingly, we found very high correlation of gene coexpression profiles among the F-type ATP synthase subunits, in comparison to coexpression with other unrelated gene pairs, in both *T*. *gondii* and *P*. *falciparum* transcriptome datasets (Fig 8). This finding further supports the fact that the novel ASAPs are indeed coexpressed together and are likely bona fide subunits of the unusual F-type ATP synthase enzyme from *T*. *gondii*.

## Discussion

Mitochondrial oxidative phosphorylation is an important source of ATP in most eukaryotic organisms and is facilitated by the multimeric F-type ATP synthase enzyme complex [1,2,5]. The enzyme consists of two functionally distinct F_1_ and F_O_ sectors, which act in concert to convert the electrochemical energy into mechanical energy to facilitate ATP synthesis. The F_1_ sector α_3_β_3_ catalytic core is connected to the rotary motor formed by F_O_ subunit c ring via the rotating central stalk structure [1,2,5]. The asymmetric structure of the γ subunit of the rotating central stalk induces conformational changes in the α_3_β_3_ catalytic core, which facilitates ATP synthesis. The stator structure—comprised primarily of the F_O_ subunits b, d, h and OSCP, along with other accessory subunits—helps in holding the α_3_β_3_ catalytic core in place while the central stalk rotates [17]. Thus, all subunit components and structural features of this unique enzyme are critical for efficient ATP synthesis.

In addition to the core subunit composition described above, other accessory proteins participate in assembling the V-shaped dimer form of the enzyme complex [19]. The paired arrangement of the F_1_ complex (spaced by 12 nm) was observed in *Paramecium* inner mitochondrial membrane by freeze-fracture and deep-etching studies [54]. Subsequent studies on yeast, bovine, and other species confirmed the dimeric arrangement of the F-type ATP synthase complex by BNP, atomic force microscopy, and cryo-electron microscopy [19,55,56]. In fact, the dimer form of the enzyme appears to facilitate cristae formation by the inner mitochondrial membrane [21,22,24] and is implicated in maintenance of the mitochondrial membrane potential [57]. However, the identity of the proteins responsible for dimer formation has not been ascertained in most species. In case of the yeast enzyme, subunits e, g, and k were identified as dimer specific components, and studies with genetic mutants revealed that subunits e and g are essential in dimer formation [19,20]. Orthologs for these proteins are present in mammalian species as well.

BNP and size exclusion chromatography studies on purified yeast F-type ATP synthase revealed that the average molecular size of the dimer and monomer form of the enzyme is around 1 MDa and between 500–600 kDa, respectively [19]. Given the complex structure and assembly of the enzyme, the expected number of protein subunits in an intact dimer is around 20, based on yeast and mammalian enzyme compositions [10,11]. Interestingly, while the F_1_ subunits are well conserved across various taxons, the F_O_ subunits, except c and OSCP, appear to be poorly conserved and in many instances are not readily identified from sequence. This is especially true for a variety of unicellular eukaryotes, including many free-living and parasitic protists, as evident from Kyoto Encyclopedia of Genes and Genomes (KEGG) data (kegg.jp). Comparative genomics studies revealed that orthologs for all yeast and mammalian F_O_ subunits, except c and OSCP, are missing in the entire apicomplexan phylum [25–27]. In fact, along with *Apicomplexa*, this appears to be the case for all other taxons belonging to the Alveolata infrakingdom. A study on the subunit composition for F-type ATP synthase from the ciliate *T*. *thermophila*, an alveolate species, resulted in the identification of 13 novel proteins [8]. Surprisingly, these proteins were unique to ciliates, indicating the possible existence of a unique set of F_O_ components in other alveolate species as well.

The *P*. *falciparum* enzyme was identified in dimer form with a molecular size of >1 MDa, confirming the presence of novel F_O_ subunits [26]. This study also revealed that the enzyme is essential in *P*. *falciparum*. However, in the case of *Plasmodium berghei*, F_1_ subunit β was found to be essential for development in mosquito but not for survival of blood stage parasites in mammalian host [58]. Studies with metabolic mutants in *T*. *gondii* have revealed that mitochondrial oxidative phosphorylation is an important source of ATP, especially during glucose deprivation and in the absence of a glycolytic flux [34,35]. Mitochondrial ATP synthesis is also likely essential for formation and maintenance of tissue cyst forms of *T*. *gondii* in infected hosts. Despite the importance of F-type ATP synthase in apicomplexan parasites, not much is known about its subunit composition, structure, and function in these parasites.

In this study, we have successfully identified the subunit constituents of F-type ATP synthase from *T*. *gondii*, a model apicomplexan parasite. Intact monomer and dimer forms of the enzyme can be identified by BNP analysis from detergent-solubilized parasite mitochondria preparations. We have identified 20 novel proteins (ASAPs) as subunit constituents of the *T*. *gondii* F-type ATP synthase by LC-MS/MS analysis of partially purified enzyme using BNP, IP, and chromatographic techniques. While some of these proteins could be counterparts of the missing F_O_ subunits, others are most likely accessory proteins required for dimer formation. Importantly, we were able to identify putative F_O_ subunits a (TGME49_310360), b (TGME49_231410), and d (TGME49_268830) based on conserved structural features. Although the F_O_ subunit a from yeast is known to have 6 transmembrane domains, the *T*. *gondii* protein has only 3. Nevertheless, the critical arginine residue, which is important for proton translocation [14], appears to be conserved (Fig 6B). The hallmark feature of F_O_ subunit b from yeast and other species is the presence of an extended helix that spans the distance between the membrane and the α_3_β_3_ catalytic core. Although there was very poor sequence conservation, very high structural similarity (>97% probability in HHPred assignment) can be found between yeast and *T*. *gondii* F_O_ subunit b proteins. F_O_ subunit d has no transmembrane domain and is known to interact with F_O_ subunit b via parallel/antiparallel coiled coil helical domains [2]. The putative *T*. *gondii* F_O_ subunit d was highly similar in secondary structure features over the conserved regions with the bovine enzyme. Further follow up work on the 3D structure of the enzyme is needed to verify these findings.

All F_1_ subunits and F_O_ c and OSCP subunits, along with 18 ASAPs, were found to be essential in *T*. *gondii* in a genome-wide clustered regularly interspaced short palindromic repeat (CRISPR)/CRISPR-associated 9 (Cas9)-mediated gene-knockout screen [50] (Fig 7). In the same study, 5 ASAPs were localized to the mitochondrion. We interrogated the mitochondrial localization for all subunits of *T*. *gondii* F-type ATP synthase by first considering evidence from in silico prediction of mitochondrial localization signals, followed by experimental localization of selected ASAPs (Fig 7). Further, we found that the transcript coexpression for the subunits of F-type ATP synthase was highly correlated, independently in both *T*. *gondii* and *P*. *falciparum* transcriptome datasets (Fig 8), thus providing additional evidence for functional interaction between these proteins across apicomplexan parasite taxons. In summary, evidence from gene essentiality, subcellular localization, and transcript coexpression together provide independent levels of support for our identification of ASAPs as bona fide subunits of *T*. *gondii* F-type ATP synthase. Moreover, an independent and parallel study on the subunit composition of *T*. *gondii* F-type ATP synthase [59] identified 13 novel proteins, 10 of which are among the ASAPs reported here, thus further authenticating their association with the enzyme.

As can be expected for such an important protein complex playing a fundamental biological role, phylogenetic analysis revealed that most ASAPs identified from *T*. *gondii* are conserved across the phylum Apicomplexa. This suggests that the origin of a divergent F-type ATP synthase may predate the origin of apicomplexan parasites. In support of this conclusion, we find that many of the apicomplexan ASAPs are also conserved in two other alveolate phyla, Chromerida and Dinoflagellata. This scenario is intriguing from an evolutionary perspective, since the ciliates, which are basal alveolates, have a completely different and unique set of ASAPs [8].

Moreover, the mitochondrial genome- and organelle-associated metabolic functions have undergone extensive diversification and streamlining within the alveolate infrakingdom [27], with ciliates being distinct from others. For example, the ciliate *T*. *thermophila* mitochondrial genome is approximately 47 kb and encodes 45 protein-coding genes, including the F-type ATP synthase F_O_ subunits c and a [8,60]. In contrast, the mitochondrial genomes of other alveolates—from phyla Apicomplexa, Chromerida, and Dinoflagellata—are highly reduced and encode only 3 (sometimes 2) protein-coding genes, none of which are F-type ATP synthase subunits [61–63]. This dramatic difference between the ciliates and other alveolates might signify an important bottleneck during evolutionary radiation within the alveolate infrakingdom. The origin of novel ASAPs in the common ancestor of Apicomplexa, Chromerida, and Dinoflagellata appears to have occurred following (or as a consequence of) this evolutionary bottleneck, either from de novo evolution or via lateral gene transfer.

It is striking that apicomplexan ASAPs are conserved across all plastid-bearing alveolate clades, suggesting a probable link between their origin and the ancestral red algal endosymbiont. The evolutionary history of the alveolate plastid, however, appears to be complex, with recent studies supporting serial higher order endosymbiotic events [64,65] rather than a single ancestral endosymbiotic event and subsequent vertical descent [66]. Regardless, the endosymbiont must have had its own mitochondrion and associated F-type ATP synthase, possibly constituted by the novel ASAPs. It is conceivable that, following the lateral transfer of genes from the endosymbiont genome into the host genome, the endosymbiont-derived ASAPs were targeted to the host mitochondrion and subsequently retained.

The conservation of apicomplexan ASAPs in other algal alveolates, such as the chromerids and *Perkinsus*, opens up the opportunity for studying the structure and function of this unusual enzyme from free-living alveolate species, from which the enzyme can be isolated in native conditions at higher yields and purity. Moreover, a deeper understanding of the structure and function of this enzyme will facilitate the discovery of novel antiparasitic compounds with pan-apicomplexan effect.

## Methods

### Sequence analysis and ortholog identification

#### Molecular reagents and methods

Genomic DNA and total RNA from tachyzoite stage *T*. *gondii* and plasmid DNA from recombinant *E*. *coli* (DH5α) were isolated using Qiagen kits (Germany); cDNA was prepared from total RNA using the reverse transcription kit from Thermo Fisher Scientific (United States of America). The manufacturer’s protocol was followed while using the various kits. PCR was done using a proof reading DNA polymerase obtained from Takara Bio (Japan), and all primers used in this study (S3 Table) were obtained from Integrated DNA Technologies (USA). Ligase and various restriction endonucleases were purchased from New England BioLabs (USA) or Promega (USA).

#### Plasmid constructs

Various PCR products were cloned into the Topo 2.1 plasmid backbone (Thermo Fisher Scientific, USA) and sequenced before subcloning into other plasmids used for either modification of target genomic loci or ectopic expression of full-length cDNA for gene of interest in *T*. *gondii*. The plasmid construct used for tagging the 3′ end of F-type ATP synthase subunits β (*Tg*atpβ) and oscp (*Tg*atposcp) genes with YFP-HA tag had the following features: 2,606 bp (BglII and AvrII) and 1,831 bp (BamHI and AvrII) PCR fragments amplified from the 3′ end of *Tg*atpβ and *Tg*atposcp loci, respectively, excluding the stop codon, were cloned into the Topo 2.1 plasmid, upstream of a YFP-HA tag coding sequence, followed by the 3′ UTR of the *T*. *gondii* dihydrofolate reductase–thymidilate synthase (*Tg*dhfr-ts) gene [67]. A modified pBluescript plasmid backbone was used for ectopic expression of full-length cDNA of selected *T*. *gondii* genes encoding the novel F-type ATP synthase subunits identified from this work. The cDNAs were cloned as BamHI and XbaI/NheI fragments in frame to a HA tag for constitutive expression using the *T*. *gondii* β-tubulin gene promoter and *Tg*dhfr-ts gene 3′ UTR. For isolating clonal lines of transgenic parasites, the respective plasmid constructs include either the DHFR cassette (for endogenous tagging; expresses a mutant version of the *Tg*dhfr cDNA that confers pyrimethamine resistance [68]) or the CAT cassette (for ectopic expression; expresses the chloramphenicol acetyl transferase gene, which confers resistance to chloramphenicol [69]).

#### *T*. *gondii* culture and genetic manipulation

Tachyzoite stage of *T*. *gondii* parasites (Type-I RH [RH] and Type-I RH ΔKu80 [RHΔKu80] [70]) strains were propagated following published protocols [71]. Briefly, human foreskin fibroblasts (HFFs), which were used as host cells for parasite infection, were grown in Dulbecco’s Modified Eagle Medium (DMEM High glucose) supplemented with 10% heat-inactivated fetal bovine serum, 2 mM GlutaMAX, 25 mM HEPES, and 50 μg/ml Gentamicin. All cell culture grade reagents were procured from Thermo Fisher Scientific, USA. The HFF cells were maintained at 37 °C in a humidified atmosphere containing 5% CO_2_. *T*. *gondii* tachyzoites were propagated by inoculating approximately 10^5^ freshly isolated tachyzoites into T25 flasks containing 2-week-old confluent HFF cell monolayer. The culture medium used for parasite growth is similar to that used for host cell growth except that it lacks serum. Parasites are harvested 48 hours after infection by scrapping the host cell monolayer, physically disrupting the HFF cell suspension by passing it repeatedly through a 22-gauge needle, and filtering it through a 3 μM nucleopore membrane (Whatman, GE Healthcare, USA) to obtain a suspension of parasites devoid of HFF cell debris. The number of parasites present in 1 ml of suspension is estimated from cell counts obtained using a hemocytometer.

For generating transgenic parasites expressing the *Tg*ATPβ and *Tg*ATPOSCP proteins from the endogenous loci, the RHΔKu80 parasites were transfected [70] with the respective tagging plasmids. Transfection was achieved by electroporating 10^7^ freshly harvested tachyzoite-stage parasites resuspended in 400 μl parasite culture medium containing 50 μg of linearized sterile plasmid DNA, using a BioRad (USA) Gene Pulser system using 10 μF capacitance, ∞ ohms resistance, and 1.5 kV voltage settings. Transfected parasites were immediately inoculated into a T25 flask containing HFF monolayer and allowed to invade and replicate for 12–15 hours before beginning drug selection with 1 μM pyrimethamine. For ectopic expression of cDNA with HA tags for selected genes, RH parasites were transfected with the respective plasmid constructs, and stable transformants were selected using 20 μM chloramphenicol. Clonal lines of stable transgenic parasites were isolated using the limiting dilution technique [71].

#### Measurement of intracellular ATP

For this assay, freshly isolated extracellular tachyzoite-stage parasites were incubated in culture media that either contained 5.5 mM glucose and 4 mM glutamine or 0 mM glucose and 4 mM glutamine, in order to evaluate the ability of the parasites to maintain ATP homeostasis. The antiparasitic drug atovaquone [43], an inhibitor of mtETC, was used to specifically inhibit mitochondrial ATP synthesis. After 2 hours of incubation, total cellular ATP was measured using the ViaLight Plus Cell Proliferation and Cytotoxicity Bioassay Kit (Lonza, Switzerland) as per the manufacturer’s protocol. The luminescence readout was quantified using the Varioskan Flash plate reader (Thermo Fisher Scientific, USA).

#### Microscopy and western blotting

For visualizing the expression and subcellular localization of selected ASAPs fused to a reporter tag, replicating intracellular tachyzoite-stage parasites stably expressing these proteins were imaged by microscopy. Before imaging, the parasite-infected HFF monolayers cultured on glass coverslips were washed with 1× phosphate-buffered saline (PBS) and fixed using 4% paraformaldehyde for 20 minutes before again washing and mounting on glass slides using the fluoroshield reagent (Sigma, USA). To counterstain the parasite mitochondrion, the cells were treated with 250 nM Mitotracker Red (Thermo Fisher Scientific, USA) for 20 minutes before fixing. The slides were imaged using the 63× oil immersion objective fitted to the Axio Observer inverted fluorescent microscope (Carl Zeiss, Germany), and images were processed using the Zen software (Carl Zeiss, Germany). Intrinsic fluorescence from YFP and Mitotracker Red were visualized using the excitation/emission filter combination of 493/520 and 578/599, respectively. HA-tagged proteins were visualized by immunofluorescence staining, using rabbit α-HA primary antibodies (1:1,000) followed by Alexa 488 conjugated goat anti rabbit secondary antibody (1:1,000), both purchased from Thermo Fisher Scientific, USA. After fixing, the cells were permeabilized for 5–10 minutes using 0.25% Triton X-100 in 1× PBS. They were then treated with 2% fetal bovine serum in 1× PBS for 30 minutes, followed by primary antibody for 1 hour, 3× wash with 1× PBS containing 0.25% Triton X-100, and secondary antibody for 1 hour. Finally, the cells were washed 3× with 1× PBS containing 0.25% Triton X-100, followed by a water wash, before mounting the cover slip on glass slides for imagining.

For western blotting, purified mitochondria were solubilized in 1× Laemmli buffer (120 mM Tris-HCL pH 6.8, 2% SDS, 10% glycerol, and 0.01% w/v bromophenol blue) and resolved by SDS-PAGE, followed by electro-transfer to PVDF membrane (using Tris-glycine buffer pH 8 containing 20% methanol) at 65 mA for approximately 1 hour and 20 minutes. The blots were kept in blocking buffer (5% skimmed milk in 1× PBS) overnight at 4 °C and then treated with primary antibody (1:5,000; rabbit α-HA monoclonal antibody, Thermo Fisher Scientific, USA) for 1 hour at ambient temperature. This was followed by 3× wash with 1× PBS containing 0.1% Tween 20, before incubating with horseradish peroxidase–coupled secondary antibody (1:5,000; donkey anti-rabbit antibody, Nif 824 GE Healthcare, USA) for 1 hour at ambient temperature, and 3× wash with 1× PBS containing 0.1% Tween 20. The blot was then developed using either the 3,3’-diaminobenzidine substrate (Sigma, USA) or the chemiluminescent ECL western blotting kit (GE Healthcare, USA).

#### Preparation of mitochondria from *T*. *gondii*

Freshly harvested tachyzoite-stage transgenic parasites (approximately 10^9^) expressing either *Tg*ATPβ-YFP-HA or *Tg*ATPOSCP-YFP-HA were washed with PBS and resuspended in 1–2 ml of hypotonic lysis buffer (15 mM phosphate buffer pH 7.5 and 2 mM Glucose). The cells were lysed by sonication in an iced-water bath for 30 minutes, and samples were centrifuged at 2,000 g for 2 minutes at 4 °C to remove unbroken cells and large cell debris. The supernatant was recovered into a new tube and centrifuged at 21,000 g for 15 minutes at 4 °C to pellet the mitochondria, which was then resuspended in 500 μl of mitochondria storage buffer (320 mM sucrose, 1 mM EDTA, 10 mM Tris pH 7.4). Total protein in the mitochondria suspension was estimated by Bradford method [72], and the lysate was stored in −80 °C until further use. When required, the mitochondria were recovered from storage buffer by centrifuging at 21,000 g for 15 minutes at 4 °C and lysed using mitochondria solubilization buffer A (MSB-A; 50 mM NaCl, 50 mM Imidazole, 2 mM 6-Aminohexoanic acid, 1 mM EDTA, pH 7.0) containing β-dodecyl maltoside (DDM) detergent in total protein to detergent ratio of 1:5. Solubilization was allowed to continue overnight with rocking at 4 °C, and the solubilized fraction was separated by centrifuging at 100,000 g for 20 minutes at 4 °C.

#### BNP separation, in-gel ATPase activity assay, and western blotting

In order to identify the monomeric and dimeric forms of the *T*. *gondii* F-type ATP synthase, the solubilized mitochondrial lysate was subject to one-dimensional BNP analysis [73,74]. Samples were prepared by adding glycerol to a final concentration of 5%, and Coomassie Blue G 250 5% (w/v) dye was added such that DDM detergent to dye ratio was 8:1. Samples were separated on a 3%–12% native PAGE (Thermo Fisher Scientific, USA) at 150 V setting for 30 minutes in cathode buffer A, which was then switched to cathode buffer B, and separation was continued till the blue dye exited the gel. The gel was visualized under white light to identify the various dye stained bands. Imidazole (25 mM, pH 7.0) was used as the anode buffer, while the composition of cathode buffers A and B were 50 mM Tricine, 7.5 mM imidazole pH 7.0, 0.02% Coomassie Blue G 250 dye and 50 mM Tricine, 7.5 mM imidazole pH 7.0, 0.002% Coomassie Blue G 250 dye, respectively.

For in-gel ATPase assay, after sample separation by BNP, the gel was incubated overnight in assay buffer (35 mM Tris-HCL pH 7.8, 270 mM glycine, 14 mM MgSO4, 0.2% Pb(NO_3_)_2_, and 8 mM ATP). The gel was then washed with water, and the ATPase activity of F-type ATP synthase was observed by the formation of a milky white precipitate, visible against a black background, at the region corresponding to the expected monomeric and dimeric protein bands. For western blotting after BNP separation, proteins were electro-transferred onto a PVDF membrane using Tris-glycine buffer pH 8.0 without methanol at 65 mAh for approximately 1 hour and 20 minutes. The membrane was then fixed in 8% acetic acid for 15 minutes, air dried at room temperature for 30 minutes, and washed with methanol several times to remove the Coomassie dye stain [26]. Further steps were similar to that described above for SDS PAGE western blotting.

#### IP of F-type ATP synthase complex

The supernatant from solubilized mitochondria preparation from tachyzoite stage transgenic *T*. *gondii* expressing YFP-HA tagged *Tg*ATPOSCP subunit was used for immunoprecipitating the F-type ATP synthase using α-HA antibodies (Thermo Fisher Scientific, USA). The antibodies were first cross-linked to Protein A/G Plus agarose beads (Santa Cruz Biotechnology, USA) using the following protocol. Approximately 50 μl of Protein A/G Plus agarose beads were washed twice in 500 μl 1× PBS (pH 7.4) at 4 °C and mixed with 1 ml of 1× PBS containing 5–7 μg of rabbit α-HA antibody and incubate overnight at 4 °C with gentle mixing on rocker. The beads were then separated by centrifugation (1,000 g for 2 minute at 4 °C), equilibrated with 0.2 M Triethanolamine (pH 8.2) for 2 minutes, and then washed twice with the same buffer. The bound antibodies were then cross-linked to the beads using 20 mM DMP (Sigma, USA) in 1 ml of 0.2 M Triethanolamine at room temperature for 45 minutes, followed by washing with 1 ml of 50 mM Tris pH 7.5 twice for 15 minutes to quench the cross-linking reaction. Unbound antibodies are removed using 3 quick washes with 0.2 M glycine buffer pH 2.3. The antibody conjugated beads were then washed twice with 1× PBS and equilibrate with MSB containing the detergent DDM at critical micelle concentration. The supernatant from solubilized mitochondria was added to the antibody coupled beads and incubated overnight at 4 °C with gentle mixing on rocker. The beads were then washed 3 times with 1× PBS containing DDM at critical micelle concentration. Proteins captured by the antibodies were eluted in 100 μl of 0.2 M glycine buffer (pH 2.3) in 3 rounds, and the pooled eluate was neutralized with 1 M Tris (pH 8.0) and concentrated using a 3 KDa cut-off concentrator (Amicon Millipore, Germany). The samples were equilibrated with 0.1% RapiGest (Waters, USA) in 50 mM ammonium bicarbonate buffer in preparation for LC-MS analysis.

#### Purification of native F-type ATP synthase by chromatography

We followed a protocol that was previously reported for the purification of the F-type ATP synthase from the *Polytomella* sp. [75]. About 13 mg (wet weight) of mitochondrial preparation from transgenic *T*. *gondii* expressing YFP-HA tagged *Tg*ATPβ subunit was solubilized overnight in 3 ml of mitochondrial solubilization buffer B (MSB-B; 50 mM Tris HCl [pH 8.0], 1 mM MgCl_2_, and DDM at protein to detergent ration 1:5). The supernatant was loaded onto a HiTrap DEAE sepharose column (GE Healthcare Life sciences, USA) having a bed volume of 1 ml and equilibrated with buffer A (50 mM Tris HCl [pH 8.0] MgCl_2_ 1mM, 0.05% [w/v] DDM). After completion of loading, the column was first washed with 10 column volumes of buffer B_20_ (50 mM Tris HCl [pH 8.0], 1 mM MgCl_2_, 20 mM NaCl, and 0.05% [w/v] DDM) and eluted with a 10× column volume linear gradient using buffer B_20_ and buffer B_500_ (50 mM Tris HCl [pH 8.0], 1 mM MgCl_2_, 500 mM NaCl, and 0.05% [w/v] DDM) at a flow rate of 0.5 ml per minute. Fractions (500 μl) were collected and checked by western blotting using α-HA antibodies. Peak positive fractions were identified, pooled, and concentrated to a final volume of 200 μl using the Vivaspin concentrator (100 kDa cut-off; GE Healthcare Life sciences, USA) and separated by size exclusion chromatography using the Superose 6 Increase 3.2/300 column (GE Healthcare Life sciences, USA) having a bed volume of 2.4 ml. A 50 μl sample was loaded on the column previously equilibrated with buffer B_20_ and then eluted with 1.5 column volumes of the same buffer at a 50 μl flow rate. Fractions (100 μl) were collected, and positive fractions, identified by western blotting using α-HA antibodies, were pooled and concentrated using a 3 KDa cut-off concentrator (Amicon Millipore, Germany). Samples were then equilibrated with 0.1% RapiGest in 50 mM ammonium bicarbonate buffer in preparation for LC-MS analysis. All chromatography steps were carried out using the AKTApure system (GE Healthcare Life sciences, USA).

#### LC-MS proteomics

In order to identify the subunit components of the F-type ATP synthase from *T*. *gondii*, we performed LC-MS analysis on the partially purified enzyme obtained from BNP, IP, and chromatographic purification. Sample preparation for LC-MS analysis was based on a previous report [76]. The bands corresponding to the dimer and monomer forms of the protein were excised out from BNP gel, cut to small pieces, destained with 50% acetonitrile in 50 mM ammonium bicarbonate, and dehydrated with 100% acetonitrile. The gel pieces were then treated with 10 mM DTT in 50 mM ammonium bicarbonate for 45–60 minutes at 56 °C to reduce the proteins, followed by alkylation in the dark with 55 mM Iodoacetamide in 50 mM ammonium bicarbonate at ambient temperature for 45 minutes. Then, trypsinization of protein was carried out overnight at 37 °C, and peptides were extracted using 50% acetonitrile in 2% formic acid. The extracts were dried using a speedvac, and the peptides were reconstituted in 50 μl of 50 mM ammonium bicarbonate, acidified with HCl, and desalted using the C_18_ Zip tip columns (Millipore, Germany). The samples were then dried in a speedvac and stored at −80 °C until further use.

Samples obtained from IP and size exclusion chromatography were immediately equilibrated with 0.1% RapiGest as stated above. The mixtures were then heated to 80 °C and maintained at this temperature for 15 minutes. The denatured proteins were reduced by 100 mM DTT at 60 °C for 15 minutes, followed by alkylation with 200 mM iodoacetamide at ambient temperature and in the dark for 30 minutes. Samples were then treated overnight with trypsin (Sigma, USA) at 20:1 substrate to enzyme ratio at 37 °C. Trypsinization was stopped by addition of 2 μl of 1 N HCL and incubated at 37 °C for 20 minutes, after which they were vortexed and centrifuged. The peptide samples were desalted using the C_18_ Zip tip (Millipore, Germany), dried, and stored at −80 °C until further use.

The peptide samples were analyzed using LC-MS^E^ workflow on the nano ACQUITY (UPLC) Synapt system (Waters Corporation, USA). The digested peptide sample (4 μl) was injected into a 5 μm Symmetry C_18_ trapping column (180 μm × 20 mm) at a flow rate of 5 μl/minute, and peptides were eluted by using the following protocol: 3%–40% B (B composition: 100% acetonitrile with 0.1% formic acid) for 90 minutes, 40%–85% B from 90–105 minutes and 97% A (A composition: 0.1% formic acid in water), and 3% B from 105–120 minutes, on a bridge ethyl hybrid C_18_ column (75 μm × 250 mm, 1.7 μm) at flow rate of 250 nl/minute. The system was coupled to the Synapt High Definition Mass Spectrometer (Waters Corporation, USA) with a nonoLock spray as the ion source. Standard glu-fibrinopeptide B (Sigma, USA) as the lockmass calibrate peptide was infused into ion source having 500 nl/minute flow rate and sampled every 30 seconds. Acquisition of LC-MS^E^ data was performed in positive V mode with mass range m/z 50–1,900, having a scan time of 0.75 second, a constant low energy of 4 V for MS mode, and 20–40 V of collision energy during high-energy MS^E^ mode scan. A capillary voltage of 3.5 kV and cone voltage 38 V were maintained during analysis. LC-MS^E^ data were processed using Protein Lynx Global Server (PLGS Version 2.5.3, Waters Corporation, USA) for identifying the proteins, with reference to the *T*. *gondii* proteome taken from ToxoDB.org release 36.

#### Gene coexpression analysis and phylogenetic studies

Normalized signal intensity values from microarray hybridizations performed at 13 different time points in the tachyzoite stage [52] were obtained directly from EupathDB.org. Normalized microarray intensities from time series data for intraerythrocytic stage *P*. *falciparum* were retrieved from reference [53]. Pearson correlation coefficients were calculated for all gene pairs using R (R-project.org). *P*-values were calculated using the Mann-Whitney U test [77] for coexpression between ATP synthase genes versus coexpression between ATP synthase genes and non-ATP synthase genes and for coexpression between ATP synthase genes versus coexpression between non-ATP synthase genes. Since minute differences in very large samples may lead to very small *p*-values, the effect size was therefore tested using Cohen’s d test [78], for which values above 0.8 denote large effect size. Orthologs for the novel subunits of *T*. *gondii* ATP synthase were identified, as reported previously [42]. Sequences were aligned with mafft (v7.222) [79] and Neighbor-Joining trees were constructed using Clustalw (2.1) [80] using 1,000 bootstrap replicates. The expected “true” phylogeny was adopted from a previous study [42]. The tree data have been deposited in TreeBASE (TB2:S22877).

## Supporting information

S1 Fig Schematic representation for genomic locus tagging for TGME49_261950 (*Tgatpβ*) and TGME49_284540 (*Tgatposcp*) with YFP-HA epitope tag. Using the respective F and R primer pairs, the 3′ end regions for these two genes were PCR amplified without stop codon (red asterisk). After transfection, the presence of the desired genomic modification was confirmed by genomic PCRs performed using the ConF and YFP_R primer pairs for the respective genes. The two gel pictures show the results from genomic PCR amplifications confirming the endogenous tagging, which is evident from the presence and absence of the PCR products in lanes 3 (transgenic) and 6 (parental), respectively. YFP-HA, yellow fluorescent protein plus hemagglutinin.
(TIF)

S2 Fig Secondary structure prediction for F_O_ subunit b–like protein from *T*. *gondii*. (A) HHpred (Homology detection and structure predication by HHM-HHM comparison [46,47]) tool was used to identify the ASAP TGME49_231410 as the likely F-type ATP synthase subunit b. Searches were done on the PDB_mmCIF70 database [46,47] using default parameters. The top 4 best hits with greater than 95% probability were for F_O_ subunit b from *Scer*, *Btau*, *Oang*, and *Ecol*. The table provides details of the amino acid length and a probability score for the prediction from the hit alignments. The pairwise alignments are shown for a 166-amino-acid-long region of the *T*. *gondii* protein. (B) The predicted secondary structure features of TGME49_231410 in comparison to *Scer* counterpart. (C) Multiple sequence alignment of putative F_O_ subunit b protein from *T*. *gondii* with *Btau*, *Scer*, and *Oang* counterparts using Custal omega [48] and ESPript [49]. The helices shown are derived from the crystal structure of *Btau* protein. Species names: *Btau*, *B*. *taurus*; *Tgon*, *T*. *gondii*; *Scer*, *S*. *cerevisiae*; *Oang*, *Ogataea angusta*. ASAP, ATP synthase–associated protein.
(TIF)

S3 Fig Secondary structure prediction for F_O_ subunit d protein from *T*. *gondii*. A similar analysis was done as described in S2 Fig. (A) HHpred tool was used to identify the ASAP TGME49_268830 as the likely F-type ATP synthase subunit d. The three best hits with greater than 95% probability were from *Scer*, *Btau* and *Oang*. (B) Predicted secondary structure features for TGME49_268830 in comparison to *Scer* counterpart. (C) Multiple sequence alignment of putative F_O_ subunit b protein from *T*. *gondii* with *Btau*, *Scer* and *Oang* proteins. The helices shown are derived from the crystal structure of *Btau* protein. Species names: *Btau*, *B*. *taurus*; *Tgon*, *T*. *gondii*; *Scer*, *S*. *cerevisiae*; *Oang*, *O*. *angusta*.
(TIF)

S4 Fig Neighbor-Joining phylogenetic trees showing the evolutionary relationship of all orthologs of ASAPs identified from representative species of Haemosporidia, Piroplasma, Coccidia, Cryptosporidiidae, Chromerida, Dinoflagellate, Ciliophora, and outgroups. (A) Cladogram representation of the expected phylogenetic relation for the selected species as previously published [42]. I–XIII represent the nodes on the cladogram and are used to denote the monophyly of taxon-specific sequences in the individual trees for each ASAP ortholog set shown in (B). The taxon color coding is same in (A) and (B). The numbers in (B) indicate bootstrapping support. ASAP, ATP synthase–associated protein.
(PDF)

S5 Fig Confirming mitochondrial localization for selected ASAPs. The cDNA for TGME49_245450, TGME49_282180, TGME49_290030, and TGME49_223040 were constitutively expressed from a plasmid (using β-tubulin promoter) as C-terminal HA-tagged proteins in tachyzoites stage parasites. Mitochondrial localization was confirmed by colocalization with Mitotracker. Immunostaining was carried out as described in the Methods section. ASAP, ATP synthase–associated protein; HA, hemagglutinin.
(TIF)

S1 Table Details of peptides identified by LC-MS/MS analysis of partially purified F-type ATP synthase by BNP, SEC, or IP techniques. In the case of data from BNP samples, the entries are color coded to indicate whether data were obtained for dimer or monomer bands from *Tg*ATPβ-YFP-HA or *Tg*ATPOSCP-YFP-HA expressing parasite samples. See Methods section for further details. From the raw peptide data, only those peptides with a ppm error within +/− 10 (column H in S1 Table excel file), peptide score of >7 (column I in S1Table excel file), and intensity value >1,000 (column O in S1Table excel file) were used for identifying the proteins, and these are the ones listed in the table. The data for BNP, SEC, and IP are shown in sheets 1, 2, and 3 of the Excel file, respectively. BNP, blue native PAGE; IP, immunoprecipitation; LC-MS/MS, liquid chromatography–tandem mass spectrometry; SEC, size exclusion chromatography; *Tg*ATPβ, *T*. *gondii* ATP synthase β subunit; *Tg*ATPOSCP, *T*. *gondii* ATP synthase OSCP subunit; YFP-HA, yellow fluorescent protein plus hemagglutinin.
(XLSX)

S2 Table List of orthologs for all *T*. *gondii* F-type ATP synthase subunits from various species. Orthologs were identified from orthoMCL.org database. For species not included in the orthoMCL database, ortholog finding was done using the ortholog assignment tool available from the database. A dash within the cell indicates that the ortholog cannot be identified in the species in question. Gene ID shown in red font indicates that the gene was clustered in a different ortholog group (as in OrthoMCL DB release 5) from the one in which the *T*. *gondii* protein was grouped.
(XLSX)

S3 Table List of primers used in this study. Restriction enzyme sites are underlined, and all primers are shown in 5′ to 3′ orientation.
(DOCX)

S1 Data Raw data from the total cellular ATP quantification experiment using a luminescence readout. These data correspond to Fig 2D. The luminescence readout values from *wt* parental strain and *Tg*ATPβ-YFP-HA- and *Tg*ATPOSCP-YFP-HA-expressing transgenic parasites—which correspond to total cellular ATP after incubating in the presence/absence of glucose and atovaquone—are shown. The data were plotted after background correction of the log_2_ transformed values. *Tg*ATPβ, *T*. *gondii* ATP synthase β subunit; *Tg*ATPOSCP, *T*. *gondii* ATP synthase OSCP subunit; YFP-HA, yellow fluorescent protein plus hemagglutinin.
(XLSX)

## Abbreviations

ASAP: ATP synthase–associated protein
BNP: blue native PAGE
Cas9: CRISPR-associated 9
CRISPR: clustered regularly interspaced short palindromic repeat
DDM: β-dodecyl maltoside
DMEM: Dulbecco’s Modified Eagle Medium
HFF: human foreskin fibroblast
IF1: inhibitory factor 1
IP: immunoprecipitation
KEGG: Kyoto Encyclopedia of Genes and Genomes
LC-MS: liquid chromatography–mass spectrometry
LC-MS/MS: liquid chromatography–tandem mass spectrometry
MSB-A: mitochondria solubilization buffer A
mtETC: mitochondrial electron transport chain
OD: optical density
OSCP: oligomycin sensitivity–conferring protein
PBS: phosphate-buffered saline
RHΔKu80: Type-I RH ΔKu80
*Tg*ATPOSCP: *T*. *gondii* ATP synthase OSCP subunit
*Tg*ATPβ: *T*. *gondii* ATP synthase β subunit
*Tg*dhfr-ts: *T*. *gondii* dihydrofolate reductase–thymidilate synthase
YFP-HA: yellow fluorescent protein plus hemagglutinin

## References

[1] Capaldi RA, Aggeler R. Mechanism of the F(1)F(0)-type ATP synthase, a biological rotary motor. Trends Biochem Sci. 2002 3;27(3):154–60. 11893513 10.1016/s0968-0004(01)02051-5

[2] Devenish RJ, Prescott M, Rodgers AJ. The structure and function of mitochondrial F1F0-ATP synthases. Int Rev Cell Mol Biol. 2008;267:1–58. 10.1016/S1937-6448(08)00601-1 18544496

[3] Mitchell P. Chemiosmotic coupling in oxidative and photosynthetic phosphorylation. Biol Rev Camb Philos Soc. 1966 8;41(3):445–502. 5329743 10.1111/j.1469-185x.1966.tb01501.x

[4] Stock D, Leslie AG, Walker JE. Molecular architecture of the rotary motor in ATP synthase. Science. 1999 11 26;286(5445):1700–5. 10576729 10.1126/science.286.5445.1700

[5] Boyer PD. The ATP synthase—a splendid molecular machine. Annu Rev Biochem. 1997;66:717–49. 10.1146/annurev.biochem.66.1.717 9242922

[6] Vázquez-Acevedo M, Cardol P, Cano-Estrada A, Lapaille M, Remacle C, González-Halphen D. The mitochondrial ATP synthase of chlorophycean algae contains eight subunits of unknown origin involved in the formation of an atypical stator-stalk and in the dimerization of the complex. J Bioenerg Biomembr. 2006 12;38(5–6):271–82. 10.1007/s10863-006-9046-x 17160464

[7] Zíková A, Schnaufer A, Dalley RA, Panigrahi AK, Stuart KD. The F(0)F(1)-ATP synthase complex contains novel subunits and is essential for procyclic Trypanosoma brucei. PLoS Pathog. 2009 5;5(5):e1000436 10.1371/journal.ppat.1000436 19436713 PMC2674945

[8] Balabaskaran Nina P, Dudkina NV, Kane LA, van Eyk JE, Boekema EJ, Mather MW, et al Highly divergent mitochondrial ATP synthase complexes in Tetrahymena thermophila. PLoS Biol. 2010 7 13;8(7):e1000418 10.1371/journal.pbio.1000418 20644710 PMC2903591

[9] Yadav KNS, Miranda-Astudillo HV, Colina-Tenorio L, Bouillenne F, Degand H, Morsomme P, et al Atypical composition and structure of the mitochondrial dimeric ATP synthase from Euglena gracilis. Biochim Biophys Acta. 2017 4;1858(4):267–275. 10.1016/j.bbabio.2017.01.007 28089911

[10] Velours J, Arselin G. The Saccharomyces cerevisiae ATP synthase. J Bioenerg Biomembr. 2000 8;32(4):383–90. 11768300 10.1023/a:1005580020547

[11] Meyer B, Wittig I, Trifilieff E, Karas M, Schägger H. Identification of two proteins associated with mammalian ATP synthase. Mol Cell Proteomics. 2007 10;6(10):1690–9. 10.1074/mcp.M700097-MCP200 17575325

[12] Rühle T, Leister D. Assembly of F1F0-ATP synthases. Biochim Biophys Acta. 2015 9;1847(9):849–60. 10.1016/j.bbabio.2015.02.005 25667968

[13] Cain BD, Simoni RD. Proton translocation by the F1F0ATPase of Escherichia coli. Mutagenic analysis of the a subunit. J Biol Chem. 1989 2 25;264(6):3292–300. 2536742

[14] Valiyaveetil FI, Fillingame RH. On the role of Arg-210 and Glu-219 of subunit a in proton translocation by the Escherichia coli F0F1-ATP synthase. J Biol Chem. 1997 12 19;272(51):32635–41. 9405480 10.1074/jbc.272.51.32635

[15] Rastogi VK, Girvin ME. Structural changes linked to proton translocation by subunit c of the ATP synthase. Nature. 1999 11 18;402(6759):263–8. 10.1038/46224 10580496

[16] Diez M, Zimmermann B, Börsch M, König M, Schweinberger E, Steigmiller S, et al Proton-powered subunit rotation in single membrane-bound F0F1-ATP synthase. Nat Struct Mol Biol. 2004 2;11(2):135–41. 10.1038/nsmb718 14730350

[17] Weber J. ATP synthase: subunit-subunit interactions in the stator stalk. Biochim Biophys Acta. 2006 Sep-Oct;1757(9–10):1162–70. 10.1016/j.bbabio.2006.04.007 16730323 PMC1785291

[18] Walker JE, Dickson VK. The peripheral stalk of the mitochondrial ATP synthase. Biochim Biophys Acta. 2006 May-Jun;1757(5–6):286–96. 10.1016/j.bbabio.2006.01.001 16697972

[19] Arnold I, Pfeiffer K, Neupert W, Stuart RA, Schägger H. Yeast mitochondrial F1F0-ATP synthase exists as a dimer: identification of three dimer-specific subunits. EMBO J. 1998 12 15;17(24):7170–8. 10.1093/emboj/17.24.7170 9857174 PMC1171063

[20] Brunner S, Everard-Gigot V, Stuart RA. Su e of the yeast F1Fo-ATP synthase forms homodimers. J Biol Chem. 2002 12 13;277(50):48484–9. 10.1074/jbc.M209382200 12377768

[21] Paumard P, Vaillier J, Coulary B, Schaeffer J, Soubannier V, Mueller DM, et al The ATP synthase is involved in generating mitochondrial cristae morphology. EMBO J. 2002 2 1;21(3):221–30. 10.1093/emboj/21.3.221 11823415 PMC125827

[22] Dudkina NV, Heinemeyer J, Keegstra W, Boekema EJ, Braun HP. Structure of dimeric ATP synthase from mitochondria: an angular association of monomers induces the strong curvature of the inner membrane. FEBS Lett. 2005 10 24;579(25):5769–72. 10.1016/j.febslet.2005.09.065 16223490

[23] Dudkina NV, Sunderhaus S, Braun HP, Boekema EJ. Characterization of dimeric ATP synthase and cristae membrane ultrastructure from Saccharomyces and Polytomella mitochondria. FEBS Lett. 2006 6 12;580(14):3427–32. 10.1016/j.febslet.2006.04.097 16714019

[24] Davies KM, Anselmi C, Wittig I, Faraldo-Gómez JD, Kühlbrandt W. Structure of the yeast F1Fo-ATP synthase dimer and its role in shaping the mitochondrial cristae. Proc Natl Acad Sci U S A. 2012 8 21;109(34):13602–7. 10.1073/pnas.1204593109 22864911 PMC3427116

[25] Mather MW, Henry KW, Vaidya AB. Mitochondrial drug targets in apicomplexan parasites. Curr Drug Targets. 2007 1;8(1):49–60. 17266530 10.2174/138945007779315632

[26] Balabaskaran Nina P, Morrisey JM, Ganesan SM, Ke H, Pershing AM, Mather MW, et al ATP synthase complex of Plasmodium falciparum: dimeric assembly in mitochondrial membranes and resistance to genetic disruption. J Biol Chem. 2011 12 2;286(48):41312–22. 10.1074/jbc.M111.290973 21984828 PMC3308843

[27] Danne JC, Gornik SG, Macrae JI, McConville MJ, Waller RF. Alveolate mitochondrial metabolic evolution: dinoflagellates force reassessment of the role of parasitism as a driver of change in apicomplexans. Mol Biol Evol. 2013 1;30(1):123–39. 10.1093/molbev/mss205 22923466

[28] Weiss LM, Dubey JP. Toxoplasmosis: A history of clinical observations. Int J Parasitol. 2009 7 1;39(8):895–901. 10.1016/j.ijpara.2009.02.004 19217908 PMC2704023

[29] Dubey JP. Advances in the life cycle of Toxoplasma gondii. Int J Parasitol. 1998 7;28(7):1019–24. 9724872 10.1016/s0020-7519(98)00023-x

[30] Dubey JP, Lindsay DS, Speer CA. Structures of Toxoplasma gondii tachyzoites, bradyzoites, and sporozoites and biology and development of tissue cysts. Clin Microbiol Rev. 1998 4;11(2):267–99. 9564564 10.1128/cmr.11.2.267 PMC106833

[31] Polonais V, Soldati-Favre D. Versatility in the acquisition of energy and carbon sources by the Apicomplexa. Biol Cell. 2010 4 23;102(8):435–45. 10.1042/BC20100005 20586726

[32] Pomel S, Luk FC, Beckers CJ. Host cell egress and invasion induce marked relocations of glycolytic enzymes in Toxoplasma gondii tachyzoites. PLoS Pathog. 2008 10;4(10):e1000188 10.1371/journal.ppat.1000188 18949028 PMC2563030

[33] Al-Anouti F, Tomavo S, Parmley S, Ananvoranich S. The expression of lactate dehydrogenase is important for the cell cycle of Toxoplasma gondii. J Biol Chem. 2004 12 10;279(50):52300–11. 10.1074/jbc.M409175200 15459194

[34] Blume M, Rodriguez-Contreras D, Landfear S, Fleige T, Soldati-Favre D, Lucius R, et al Host-derived glucose and its transporter in the obligate intracellular pathogen Toxoplasma gondii are dispensable by glutaminolysis. Proc Natl Acad Sci U S A. 2009 8 4;106(31):12998–3003. 10.1073/pnas.0903831106 19617561 PMC2722337

[35] MacRae JI, Sheiner L, Nahid A, Tonkin C, Striepen B, McConville MJ. Mitochondrial metabolism of glucose and glutamine is required for intracellular growth of Toxoplasma gondii. Cell Host Microbe. 2012 11 15;12(5):682–92. 10.1016/j.chom.2012.09.013 23159057 PMC3990185

[36] Nitzsche R, Zagoriy V, Lucius R, Gupta N. Metabolic Cooperation of Glucose and Glutamine Is Essential for the Lytic Cycle of Obligate Intracellular Parasite Toxoplasma gondii. J Biol Chem. 2016 1 1;291(1):126–41. 10.1074/jbc.M114.624619 26518878 PMC4697150

[37] Vercesi AE, Rodrigues CO, Uyemura SA, Zhong L, Moreno SN. Respiration and oxidative phosphorylation in the apicomplexan parasite Toxoplasma gondii. J Biol Chem. 1998 11 20;273(47):31040–7. 9813002 10.1074/jbc.273.47.31040

[38] Saleh A, Friesen J, Baumeister S, Gross U, Bohne W. Growth inhibition of Toxoplasma gondii and Plasmodium falciparum by nanomolar concentrations of 1-hydroxy-2-dodecyl-4(1H)quinolone, a high-affinity inhibitor of alternative (type II) NADH dehydrogenases. Antimicrob Agents Chemother. 2007 4;51(4):1217–22. 10.1128/AAC.00895-06 17242151 PMC1855512

[39] Lin SS, Gross U, Bohne W. Type II NADH dehydrogenase inhibitor 1-hydroxy-2-dodecyl-4(1H)quinolone leads to collapse of mitochondrial inner-membrane potential and ATP depletion in Toxoplasma gondii. Eukaryot Cell. 2009 6;8(6):877–87. 10.1128/EC.00381-08 19286986 PMC2698307

[40] McFadden DC, Tomavo S, Berry EA, Boothroyd JC. Characterization of cytochrome b from Toxoplasma gondii and Q(o) domain mutations as a mechanism of atovaquone-resistance. Mol Biochem Parasitol. 2000 4 30;108(1):1–12. 10802314 10.1016/s0166-6851(00)00184-5

[41] Mogi T, Kita K. Diversity in mitochondrial metabolic pathways in parasitic protists Plasmodium and Cryptosporidium. Parasitol Int. 2010 9;59(3):305–12. 10.1016/j.parint.2010.04.005 20433942

[42] Woo YH, Ansari H, Otto TD, Klinger CM, Kolisko M, Michálek J, et al Chromerid genomes reveal the evolutionary path from photosynthetic algae to obligate intracellular parasites. Elife. 2015 7 15;4:e06974 10.7554/eLife.06974 26175406 PMC4501334

[43] Baggish AL, Hill DR. Antiparasitic agent atovaquone. Antimicrob Agents Chemother. 2002 5;46(5):1163–73. 10.1128/AAC.46.5.1163-1173.2002 11959541 PMC127192

[44] Pullman ME, Monroy GC. A Naturally Occurring Inhibitor Of Mitochondrial Adenosine Triphosphatase. J Biol Chem. 1963 11;238:3762–9. 14109217

[45] Krause F, Reifschneider NH, Goto S, Dencher NA. Active oligomeric ATP synthases in mammalian mitochondria. Biochem Biophys Res Commun. 2005 4 8;329(2):583–90. 10.1016/j.bbrc.2005.02.010 15737625

[46] Zimmermann L, Stephens A, Nam SZ, Rau D, Kübler J, Lozajic M, et al A Completely Reimplemented MPI Bioinformatics Toolkit with a New HHpred Server at its Core. J Mol Biol. 2017 12 16 10.1016/j.jmb.2017.12.007 29258817

[47] Söding J. Protein homology detection by HMM-HMM comparison. Bioinformatics. 2005 4 1;21(7):951–60. Erratum in: Bioinformatics. 2005 May 1;21(9):2144. 10.1093/bioinformatics/bti125 15531603

[48] Sievers F, Wilm A, Dineen D, Gibson TJ, Karplus K, Li W, et al Fast, scalable generation of high-quality protein multiple sequence alignments using Clustal Omega. Mol Syst Biol. 2011 10 11;7:539 10.1038/msb.2011.75 21988835 PMC3261699

[49] Robert X, Gouet P. Deciphering key features in protein structures with the new ENDscript server. Nucleic Acids Res. 2014 7;42(Web Server issue):W320–4. 10.1093/nar/gku316 24753421 PMC4086106

[50] Sidik SM, Huet D, Ganesan SM, Huynh MH, Wang T, Nasamu AS, et al A Genome-wide CRISPR Screen in Toxoplasma Identifies Essential Apicomplexan Genes. Cell. 2016 9 8;166(6):1423–1435.e12. 10.1016/j.cell.2016.08.019 27594426 PMC5017925

[51] Claros MG, Vincens P. Computational method to predict mitochondrially imported proteins and their targeting sequences. Eur J Biochem. 1996 11 1;241(3):779–86. 8944766 10.1111/j.1432-1033.1996.00779.x

[52] Behnke MS, Wootton JC, Lehmann MM, Radke JB, Lucas O, Nawas J, et al Coordinated progression through two subtranscriptomes underlies the tachyzoite cycle of Toxoplasma gondii. PLoS ONE. 2010 8 26;5(8):e12354 10.1371/journal.pone.0012354 20865045 PMC2928733

[53] Bozdech Z, Llinás M, Pulliam BL, Wong ED, Zhu J, DeRisi JL. The transcriptome of the intraerythrocytic developmental cycle of Plasmodium falciparum. PLoS Biol. 2003 10;1(1):E5 10.1371/journal.pbio.0000005 12929205 PMC176545

[54] Allen RD, Schroeder CC, Fok AK. An investigation of mitochondrial inner membranes by rapid-freeze deep-etch techniques. J Cell Biol. 1989 6;108(6):2233–40. 2525561 10.1083/jcb.108.6.2233 PMC2115613

[55] Buzhynskyy N, Sens P, Prima V, Sturgis JN, Scheuring S. Rows of ATP synthase dimers in native mitochondrial inner membranes. Biophys J. 2007 10 15;93(8):2870–6. 10.1529/biophysj.107.109728 17557793 PMC1989723

[56] Davies KM, Strauss M, Daum B, Kief JH, Osiewacz HD, Rycovska A, et al Macromolecular organization of ATP synthase and complex I in whole mitochondria. Proc Natl Acad Sci U S A. 2011 8 23;108(34):14121–6. 10.1073/pnas.1103621108 21836051 PMC3161574

[57] Bornhövd C, Vogel F, Neupert W, Reichert AS. Mitochondrial membrane potential is dependent on the oligomeric state of F1F0-ATP synthase supracomplexes. J Biol Chem. 2006 5 19;281(20):13990–8. 10.1074/jbc.M512334200 16551625

[58] Sturm A, Mollard V, Cozijnsen A, Goodman CD, McFadden GI. Mitochondrial ATP synthase is dispensable in blood-stage Plasmodium berghei rodent malaria but essential in the mosquito phase. Proc Natl Acad Sci U S A. 2015 8 18;112(33):10216–23. 10.1073/pnas.1423959112 25831536 PMC4547259

[59] Huet D, Rajendran E, van Dooren G, Lourido S. Identification of cryptic stator subunits from an apicomplexan ATP synthase. 2018 [cited 11 May 2018]. Preprint. bioRxiv:314385. 10.7554/eLife.38097 PMC6133553 30204085

[60] Brunk CF, Lee LC, Tran AB, Li J. Complete sequence of the mitochondrial genome of Tetrahymena thermophila and comparative methods for identifying highly divergent genes. Nucleic Acids Res. 2003 3 15;31(6):1673–82. 12626709 10.1093/nar/gkg270 PMC152872

[61] Vaidya AB, Mather MW. Mitochondrial evolution and functions in malaria parasites. Annu Rev Microbiol. 2009;63:249–67. 10.1146/annurev.micro.091208.073424 19575561

[62] Oborník M, Lukeš J. The Organellar Genomes of Chromera and Vitrella, the Phototrophic Relatives of Apicomplexan Parasites. Annu Rev Microbiol. 2015;69:129–44. 10.1146/annurev-micro-091014-104449 26092225

[63] Waller RF, Jackson CJ. Dinoflagellate mitochondrial genomes: stretching the rules of molecular biology. Bioessays. 2009 2;31(2):237–45. 10.1002/bies.200800164 19204978

[64] Petersen J, Ludewig AK, Michael V, Bunk B, Jarek M, Baurain D, et al Chromera velia, endosymbioses and the rhodoplex hypothesis—plastid evolution in cryptophytes, alveolates, stramenopiles, and haptophytes (CASH lineages). Genome Biol Evol. 2014 3;6(3):666–84. 10.1093/gbe/evu043 24572015 PMC3971594

[65] Waller RF, Gornik SG, Koreny L, Pain A. Metabolic pathway redundancy within the apicomplexan-dinoflagellate radiation argues against an ancient chromalveolate plastid. Commun Integr Biol. 2015 12 8;9(1):e1116653 10.1080/19420889.2015.1116653 27066182 PMC4802802

[66] Cavalier-Smith T. Principles of protein and lipid targeting in secondary symbiogenesis: euglenoid, dinoflagellate, and sporozoan plastid origins and the eukaryote family tree. J Eukaryot Microbiol. 1999 Jul-Aug;46(4):347–66. 18092388 10.1111/j.1550-7408.1999.tb04614.x

[67] Roos DS. Primary structure of the dihydrofolate reductase-thymidylate synthase gene from Toxoplasma gondii. J Biol Chem. 1993 3 25;268(9):6269–80. 8454599

[68] Donald RG, Roos DS. Stable molecular transformation of Toxoplasma gondii: a selectable dihydrofolate reductase-thymidylate synthase marker based on drug-resistance mutations in malaria. Proc Natl Acad Sci U S A. 1993 12 15;90(24):11703–7. 8265612 10.1073/pnas.90.24.11703 PMC48052

[69] Kim K, Soldati D, Boothroyd JC. Gene replacement in Toxoplasma gondii with chloramphenicol acetyltransferase as selectable marker. Science. 1993 11 5;262(5135):911–4. 8235614 10.1126/science.8235614

[70] Huynh MH, Carruthers VB. Tagging of endogenous genes in a Toxoplasma gondii strain lacking Ku80. Eukaryot Cell. 2009 4;8(4):530–9. 10.1128/EC.00358-08 19218426 PMC2669203

[71] Roos DS, Donald RG, Morrissette NS, Moulton AL. Molecular tools for genetic dissection of the protozoan parasite Toxoplasma gondii. Methods Cell Biol. 1994;45:27–63. 7707991 10.1016/s0091-679x(08)61845-2

[72] Bradford MM. A rapid and sensitive method for the quantitation of microgram quantities of protein utilizing the principle of protein-dye binding. Anal Biochem. 1976 5 7;72:248–54. 942051 10.1016/0003-2697(76)90527-3

[73] Wittig I, Braun HP, Schägger H. Blue native PAGE. Nat Protoc. 2006;1(1):418–28. 10.1038/nprot.2006.62 17406264

[74] Nijtmans LG, Henderson NS, Holt IJ. Blue Native electrophoresis to study mitochondrial and other protein complexes. Methods. 2002 4;26(4):327–34. 10.1016/S1046-2023(02)00038-5 12054923

[75] Allegretti M, Klusch N, Mills DJ, Vonck J, Kühlbrandt W, Davies KM. Horizontal membrane-intrinsic α-helices in the stator a-subunit of an F-type ATP synthase. Nature. 2015 5 14;521(7551):237–40. 10.1038/nature14185 25707805

[76] Korwar AM, Vannuruswamy G, Jagadeeshaprasad MG, Jayaramaiah RH, Bhat S, Regin BS, et al Development of Diagnostic Fragment Ion Library for Glycated Peptides of Human Serum Albumin: Targeted Quantification in Prediabetic, Diabetic, and Microalbuminuria Plasma by Parallel Reaction Monitoring, SWATH, and MSE. Mol Cell Proteomics. 2015 8;14(8):2150–9. 10.1074/mcp.M115.050518 26023067 PMC4528244

[77] Hollander M, Wolfe DA, Chicken E, editors. Nonparametric Statistical Methods. 3rd ed Hoboken, N.J.: Wiley; 2013.

[78] Cohen J. Statistical power analysis for the behavioral sciences. 2nd ed Hillsdale, N.J.: L. Erlbaum Associates; 1988.

[79] Katoh K, Standley DM. MAFFT multiple sequence alignment software version 7: improvements in performance and usability. Mol Biol Evol. 2013 4;30(4):772–80. 10.1093/molbev/mst010 23329690 PMC3603318

[80] Larkin MA, Blackshields G, Brown NP, Chenna R, McGettigan PA, McWilliam H, et al Clustal W and Clustal X version 2.0. Bioinformatics. 2007 11 1;23(21):2947–8. 10.1093/bioinformatics/btm404 17846036

