# Correction: Highly diverged novel subunit composition of apicomplexan F-type ATP synthase identified from *Toxoplasma gondii*

**DOI:** 10.1371/journal.pbio.3000176

**Published:** 2019-03-06

**Authors:** Rahul Salunke, Tobias Mourier, Manidipa Banerjee, Arnab Pain, Dhanasekaran Shanmugam

An affiliation is missing for Rahul Salunke and Dhanasekaran Shanmugam. These authors are also affiliated with Academy of Scientific and Innovative Research (AcSIR), Ghaziabad, India. The amended author affiliation list is included below:

Rahul Salunke,^1,**2**^ Tobias Mourier,^3^ Manidipa Banerjee^4^, Arnab Pain^3^, Dhanasekaran Shanmugam^1,**2**^

^1^ Biochemical Sciences Division, CSIR-National Chemical Laboratory, Pune, Maharashtra, India, ^**2**^ **Academy of Scientific and Innovative Research (AcSIR), Ghaziabad, India**, ^3^ Pathogen Genomics Laboratory, BESE Division, King Abdullah University of Science and Technology (KAUST), Thuwal, Saudi Arabia, ^4^ Kusuma School of Biological Sciences, Indian Institute of Technology- Delhi, Hauz Khas, New Delhi, India.
