# Supplementary figures and images for: Highly diverged novel subunit composition of apicomplexan F-type ATP synthase identified from Toxoplasma gondii
Source: PLoS Biol. 2018 Jul 13;16(7):e2006128. doi: 10.1371/journal.pbio.2006128 (PMC6059495; doi:10.1371/journal.pbio.2006128)

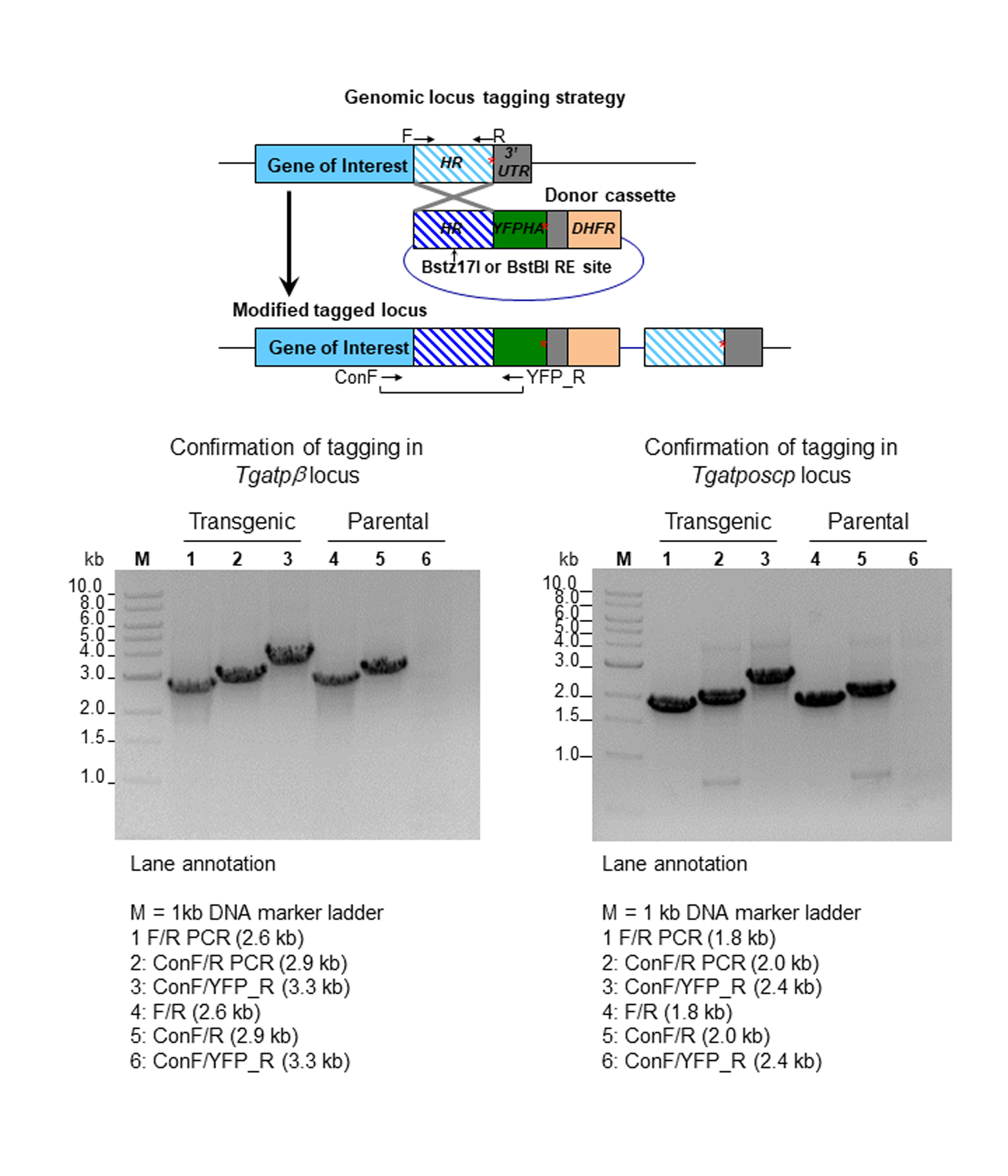

Supplement: S1 Fig — Using the respective F and R primer pairs, the 3′ end regions for these two genes were PCR amplified without stop codon (red asterisk). After transfection, the presence of the desired genomic modification was confirmed by genomic PCRs performed using the ConF and YFP_R primer pairs for the respective genes. The two gel pictures show the results from genomic PCR amplifications confirming the endogenous tagging, which is evident from the presence and absence of the PCR products in lanes 3 (transgenic) and 6 (parental), respectively. YFP-HA, yellow fluorescent protein plus hemagglutinin. (TIF) [file pbio.2006128.s001.tif]

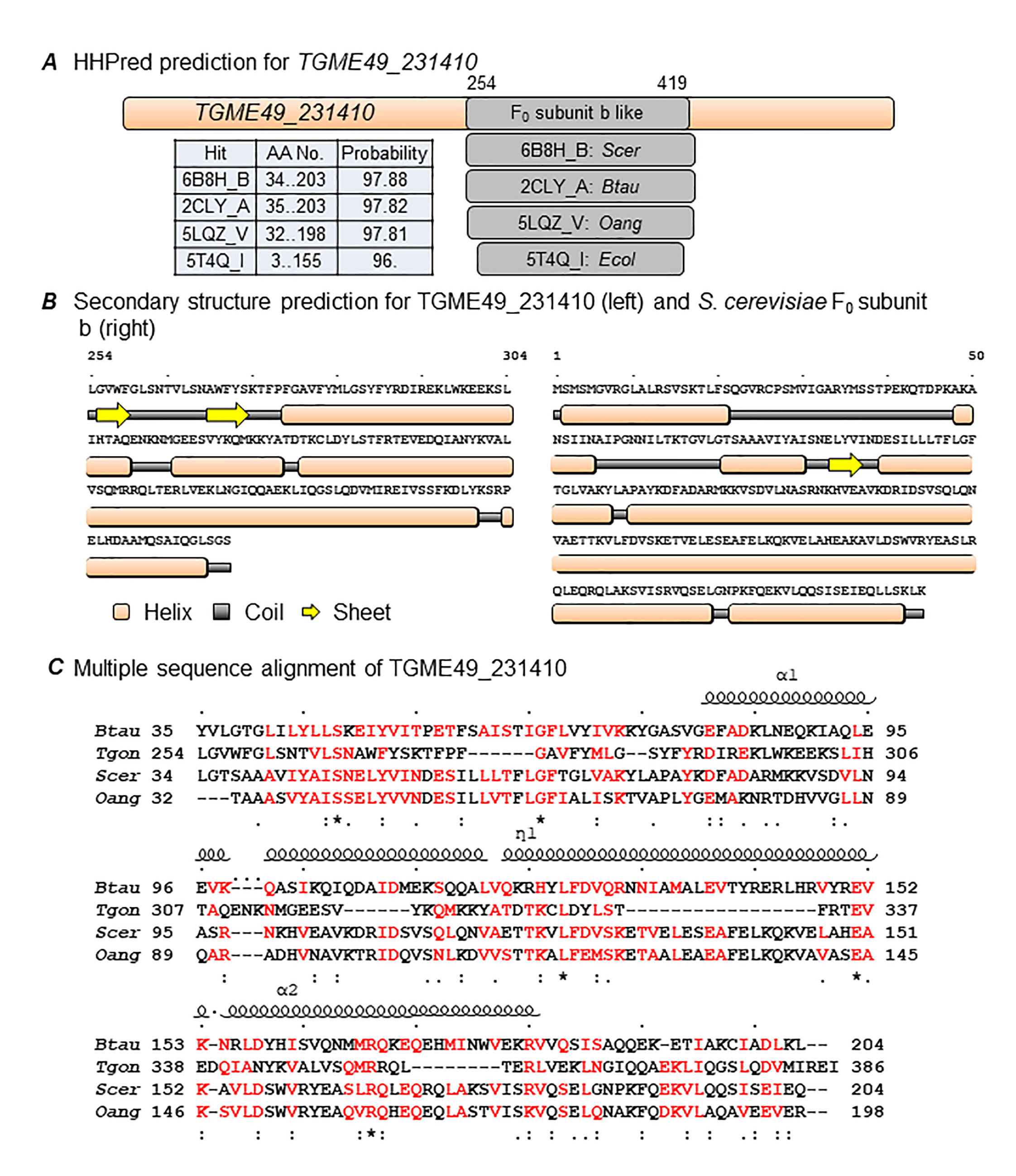

Supplement: S2 Fig — (A) HHpred (Homology detection and structure predication by HHM-HHM comparison [46,47]) tool was used to identify the ASAP TGME49_231410 as the likely F-type ATP synthase subunit b. Searches were done on the PDB_mmCIF70 database [46,47] using default parameters. The top 4 best hits with greater than 95% probability were for FO subunit b from Scer, Btau, Oang, and Ecol. The table provides details of the amino acid length and a probability score for the prediction from the hit alignments. The pairwise alignments are shown for a 166-amino-acid-long region of the T. gondii protein. (B) The predicted secondary structure features of TGME49_231410 in comparison to Scer counterpart. (C) Multiple sequence alignment of putative FO subunit b protein from T. gondii with Btau, Scer, and Oang counterparts using Custal omega [48] and ESPript [49]. The helices shown are derived from the crystal structure of Btau protein. Species names: Btau, B. taurus; Tgon, T. gondii; Scer, S. cerevisiae; Oang, Ogataea angusta. ASAP, ATP synthase–associated protein. (TIF) [file pbio.2006128.s002.tif]

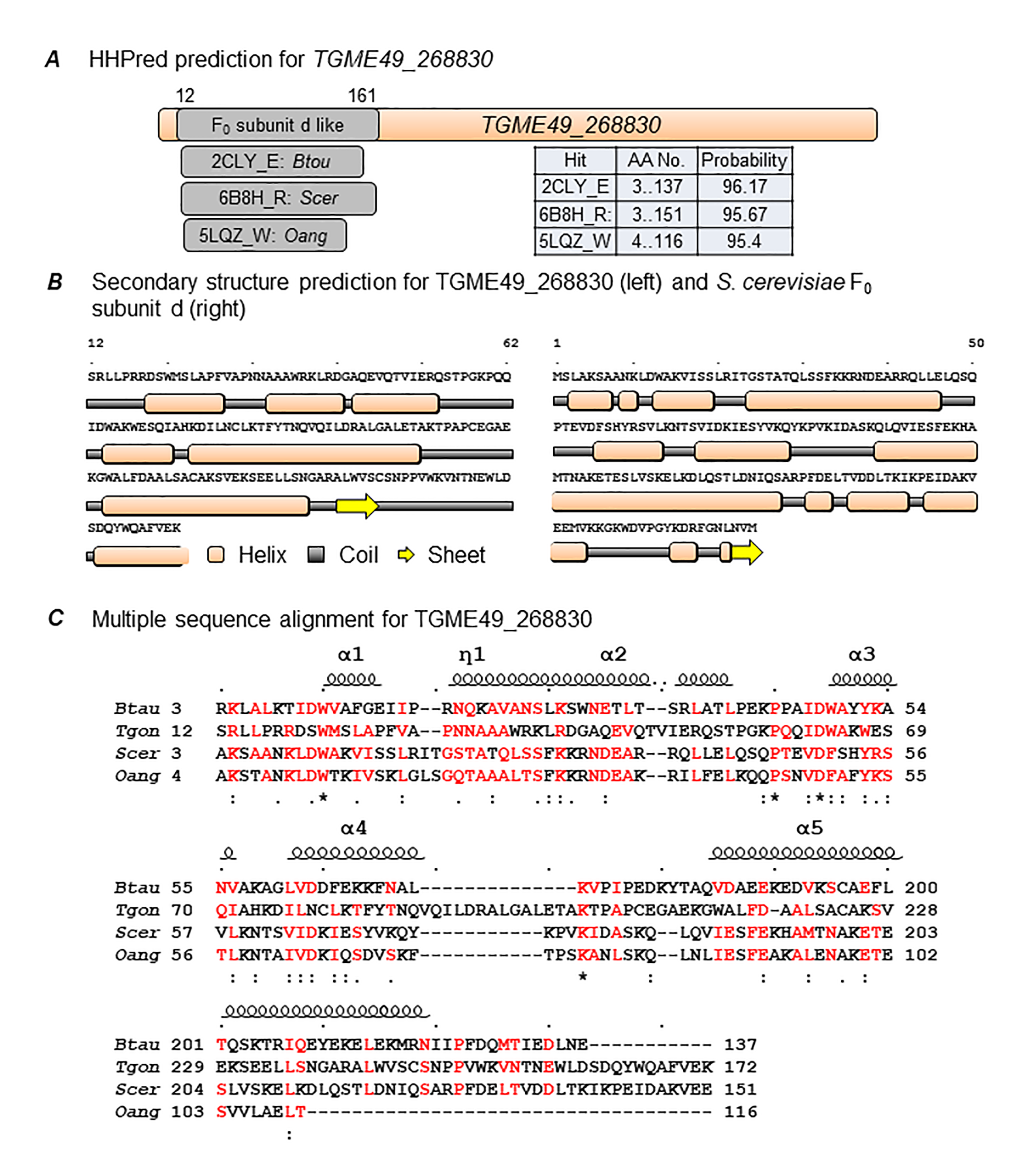

Supplement: S3 Fig — A similar analysis was done as described in S2 Fig. (A) HHpred tool was used to identify the ASAP TGME49_268830 as the likely F-type ATP synthase subunit d. The three best hits with greater than 95% probability were from Scer, Btau and Oang. (B) Predicted secondary structure features for TGME49_268830 in comparison to Scer counterpart. (C) Multiple sequence alignment of putative FO subunit b protein from T. gondii with Btau, Scer and Oang proteins. The helices shown are derived from the crystal structure of Btau protein. Species names: Btau, B. taurus; Tgon, T. gondii; Scer, S. cerevisiae; Oang, O. angusta. (TIF) [file pbio.2006128.s003.tif]

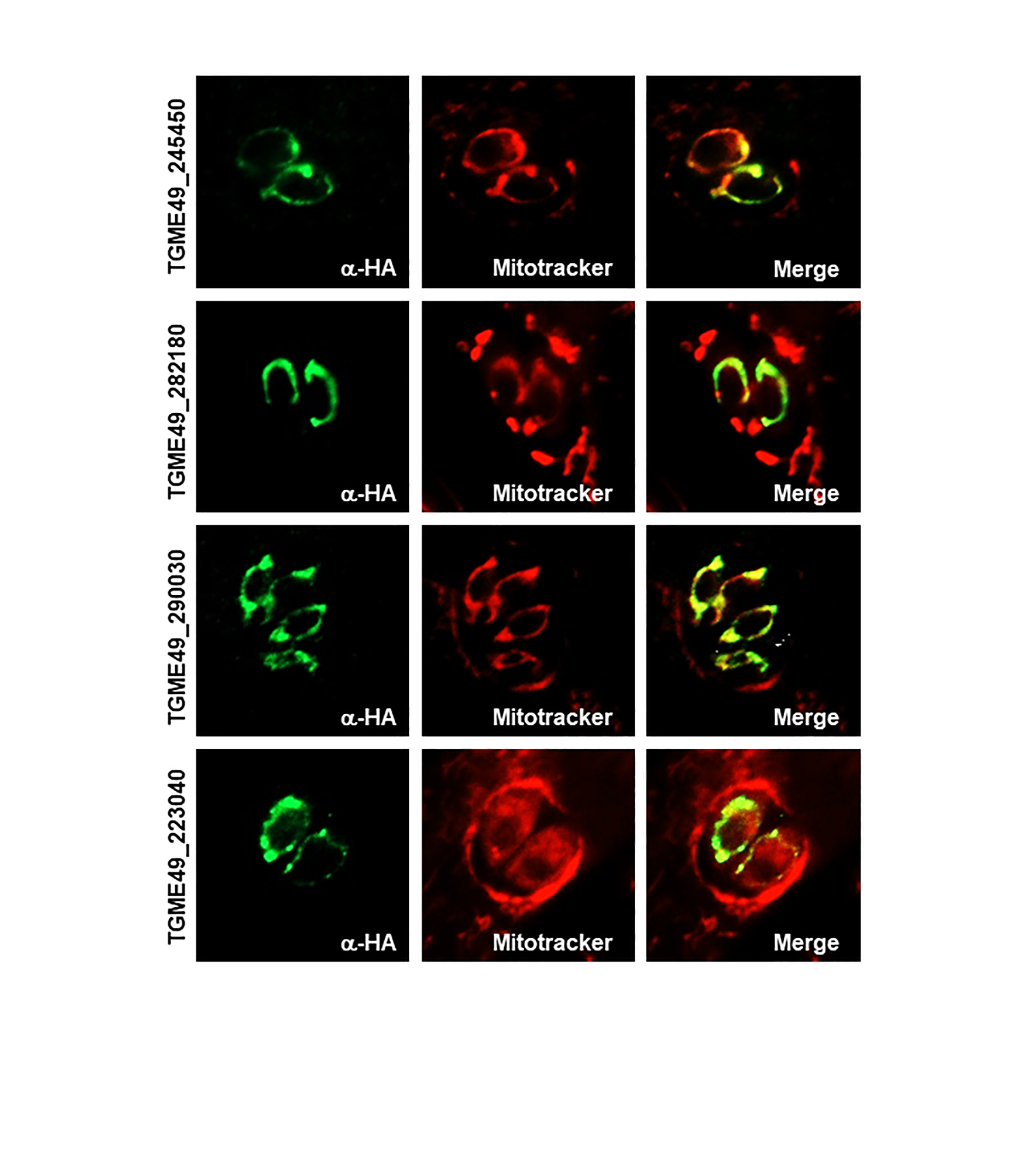

Supplement: S5 Fig — The cDNA for TGME49_245450, TGME49_282180, TGME49_290030, and TGME49_223040 were constitutively expressed from a plasmid (using β-tubulin promoter) as C-terminal HA-tagged proteins in tachyzoites stage parasites. Mitochondrial localization was confirmed by colocalization with Mitotracker. Immunostaining was carried out as described in the Methods section. ASAP, ATP synthase–associated protein; HA, hemagglutinin. (TIF) [file pbio.2006128.s005.tif]
