# Supplementary material for: Highly diverged novel subunit composition of apicomplexan F-type ATP synthase identified from Toxoplasma gondii
Source: PLoS Biol. 2018 Jul 13;16(7):e2006128. doi: 10.1371/journal.pbio.2006128 (PMC6059495; doi:10.1371/journal.pbio.2006128)
Supplement: S4 Fig — (A) Cladogram representation of the expected phylogenetic relation for the selected species as previously published [42]. I–XIII represent the nodes on the cladogram and are used to denote the monophyly of taxon-specific sequences in the individual trees for each ASAP ortholog set shown in (B). The taxon color coding is same in (A) and (B). The numbers in (B) indicate bootstrapping support. ASAP, ATP synthase–associated protein. (PDF) [file pbio.2006128.s004.pdf]

A

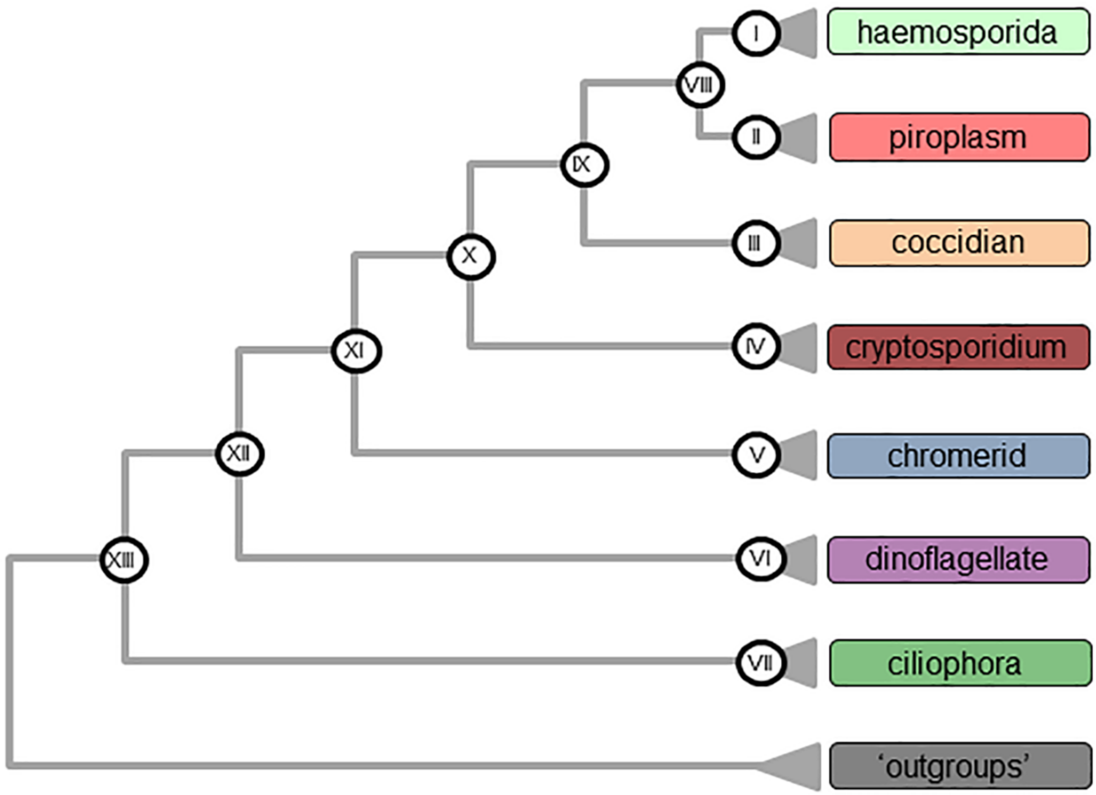
haemosporida
piroplasm
coccidian
cryptosporidium
chromerid
dinoflagellate
ciliophora
‘outgroups’
I
II
III
IV
V
VI
VII
VIII
IX
X
XI
XII
XIII

B

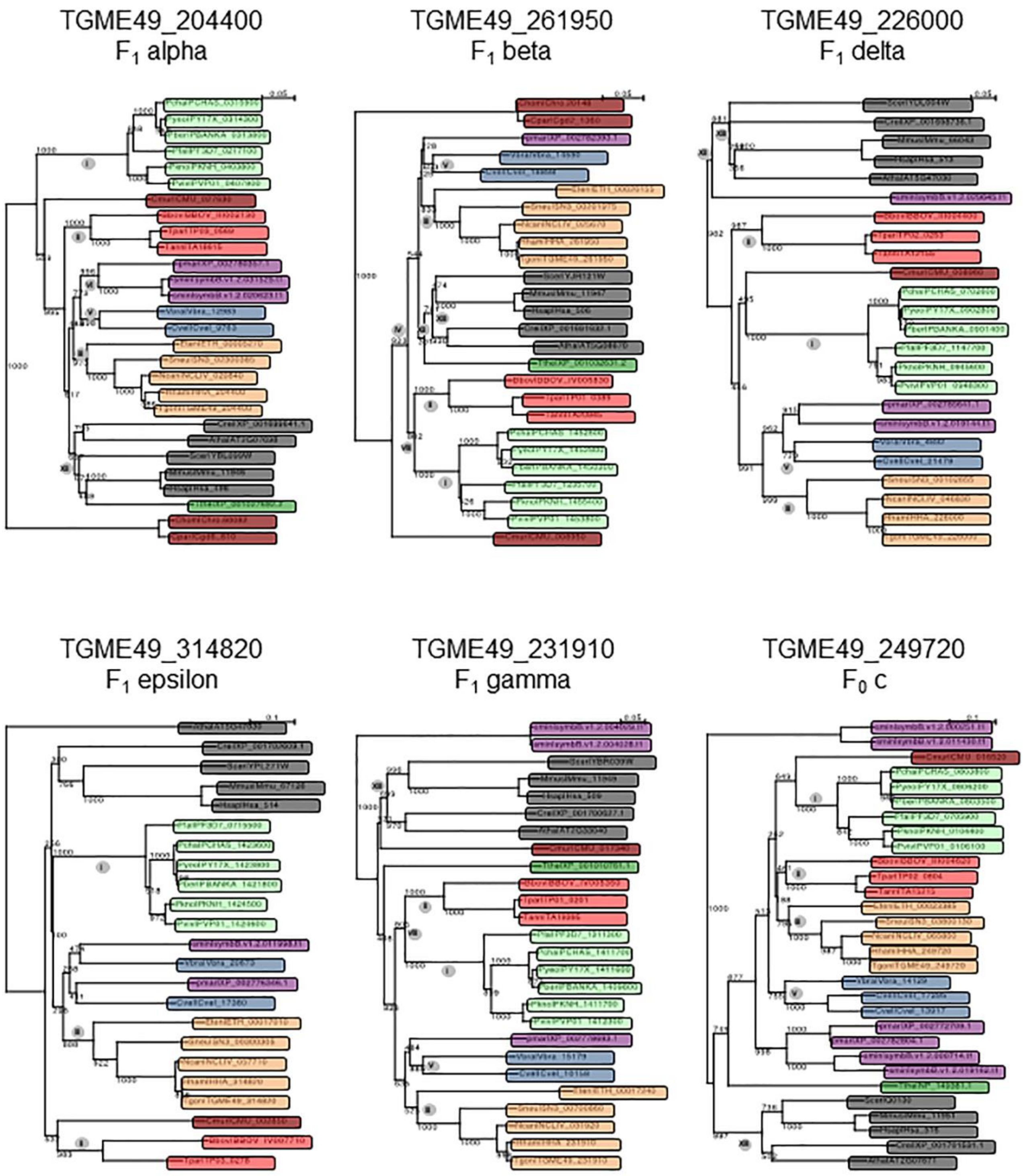

TGME49_204400
F_1 alpha
TGME49_261950
F_1 beta
TGME49_226000
F_1 delta
TGME49_314820
F_1 epsilon
TGME49_231910
F_1 gamma
TGME49_249720
F_0 c

***B** (continued)*

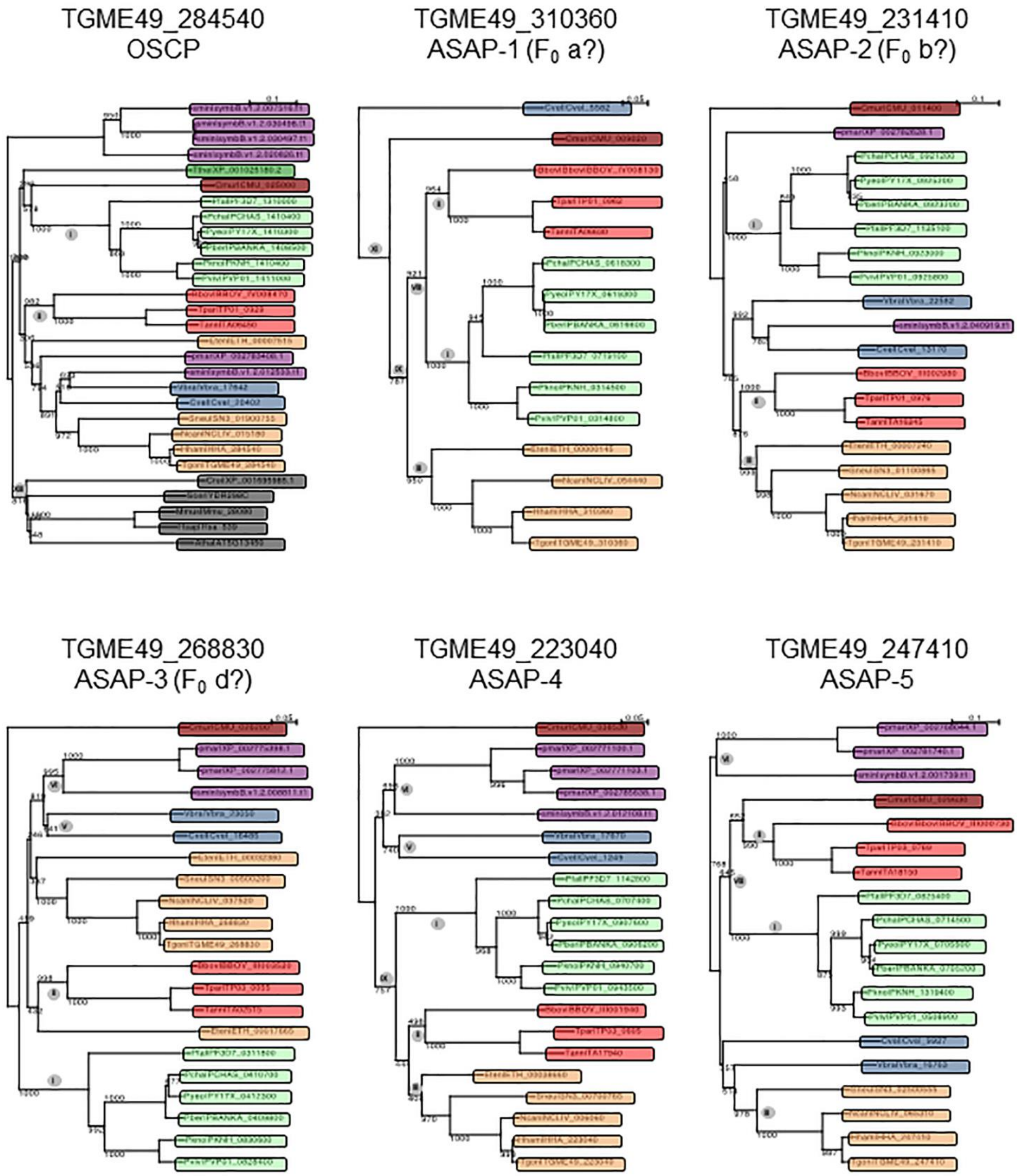
TGME49_284540
OSCP
TGME49_310360
ASAP-1 (F_0 a?)
TGME49_231410
ASAP-2 (F_0 b?)
TGME49_268830
ASAP-3 (F_0 d?)
TGME49_223040
ASAP-4
TGME49_247410
ASAP-5

B *(continued)*

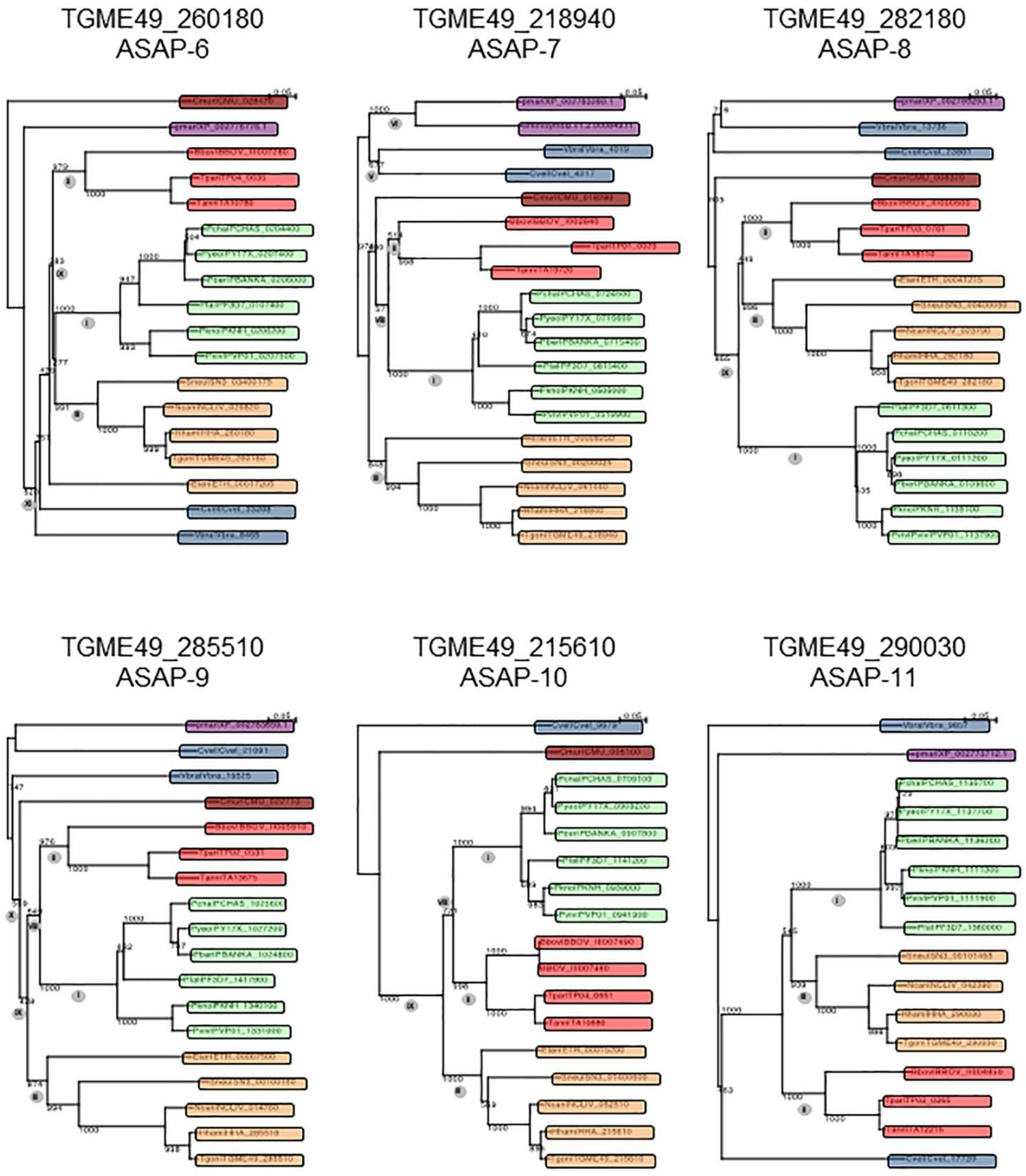
TGME49_260180
ASAP-6
TGME49_218940
ASAP-7
TGME49_282180
ASAP-8
TGME49_285510
ASAP-9
TGME49_215610
ASAP-10
TGME49_290030
ASAP-11

***B** (continued)*

TGME49_310180
ASAP-12

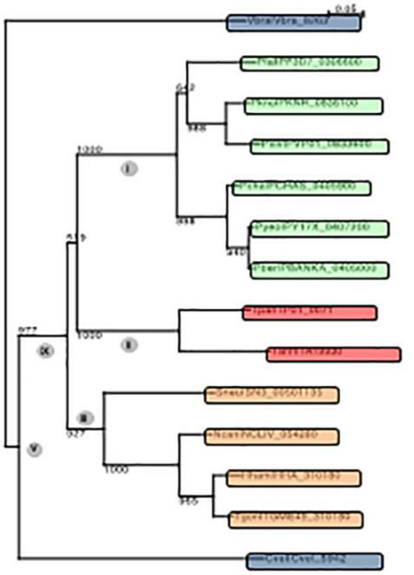

TGME49_214930
ASAP-13

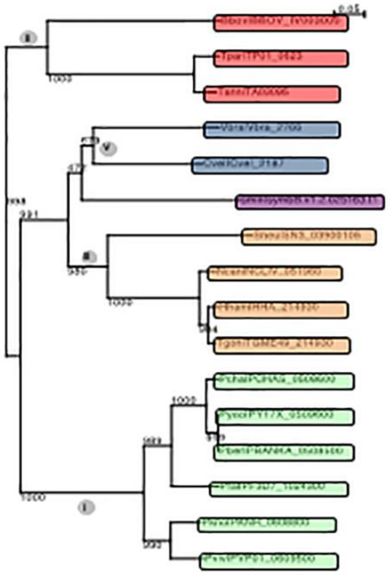

TGME49_245450
ASAP-14

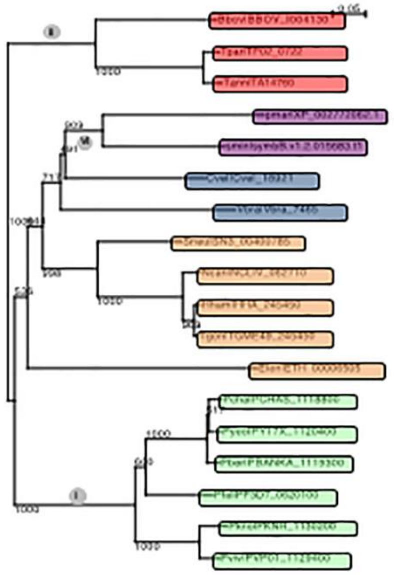

TGME49_208440
ASAP-15

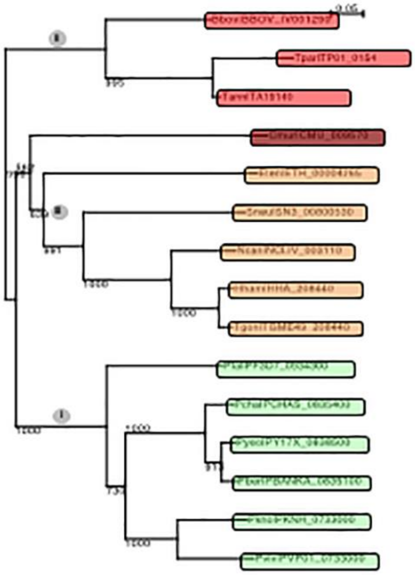

TGME49_201800
ASAP-16

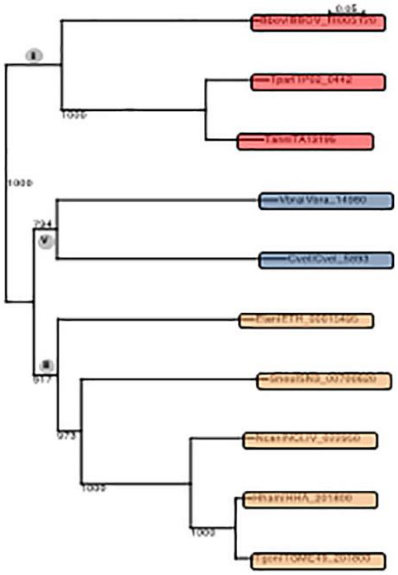

TGME49_225730
ASAP-17

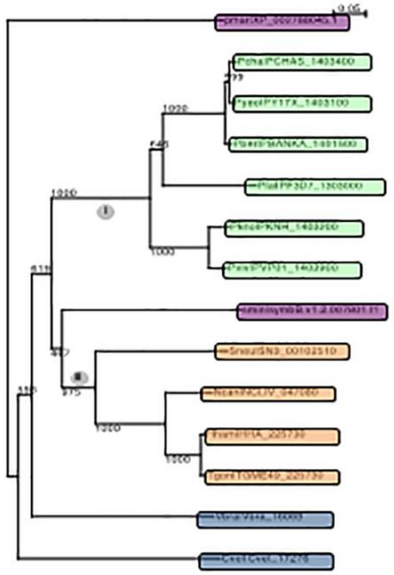

B *(continued)*

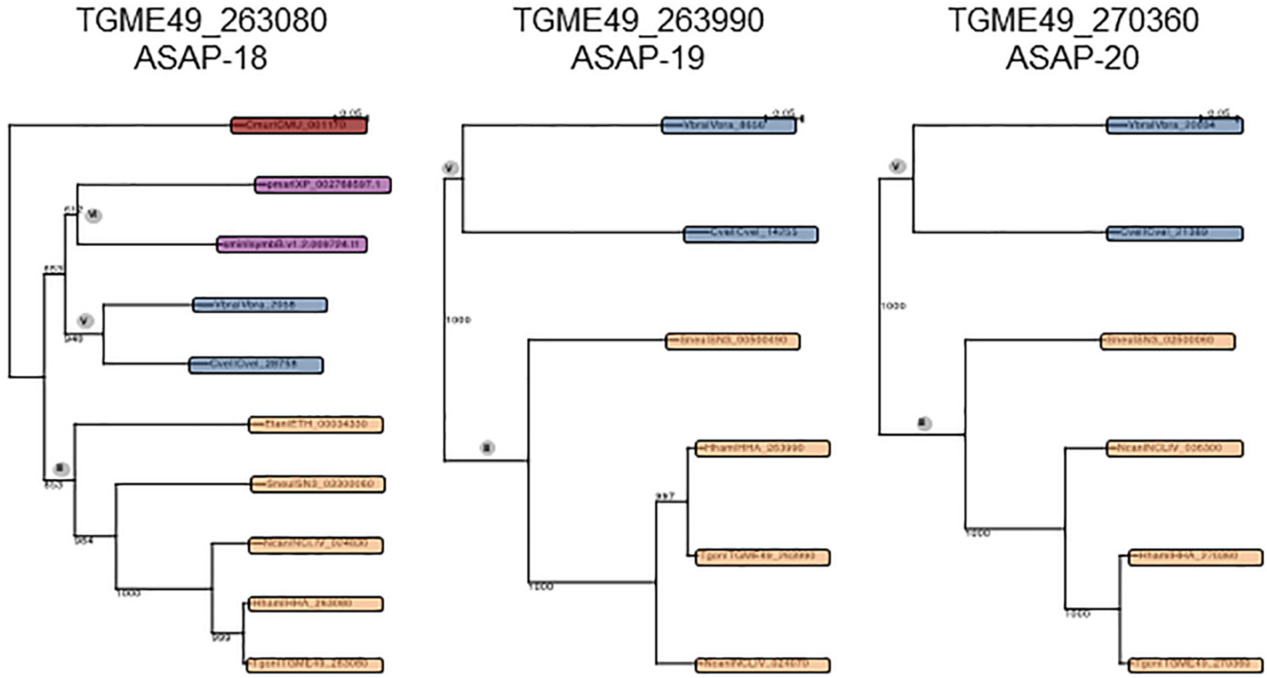
TGME49_263080
ASAP-18
TGME49_263990
ASAP-19
TGME49_270360
ASAP-20
