# Supplementary material for: Highly diverged novel subunit composition of apicomplexan F-type ATP synthase identified from Toxoplasma gondii
Source: PLoS Biol. 2018 Jul 13;16(7):e2006128. doi: 10.1371/journal.pbio.2006128 (PMC6059495; doi:10.1371/journal.pbio.2006128)
Supplement: S3 Table — Restriction enzyme sites are underlined, and all primers are shown in 5′ to 3′ orientation. (DOCX) [file pbio.2006128.s008.docx]

**S3 Table. List of Primers used in this study.** Restriction enzyme sites are underlined and all primers are shown in 5’ to 3’ orientation.

| **Primer Name** | **Primer sequence** |
| --- | --- |
| TGME49_261950_ F | TACAGATCTTCTGAACGGACTGCCTCGCG |
| TGME49_261950_ R | ACTCCTAGGCTTTCCGCTCGCCGCTTCCTGCG |
| TGME49_261950_ConF | CTGCGGCGCCGAATCCCGGAAAGAAACCAGC |
| TGME49_284540_ F | TACGGATCCGAAGCTCGCGATTTCGTGTTTCGG |
| TGME49_284540_R | ACTCCTAGGAAGAGGAGCCAGCAGCTGCGACTGC |
| TGME49_284540_ConF | CTGTGAACCACCGGGAGAAAACAACGCGGC |
| YFP_R | CCATGATATAGACGTTGTGGCTGTTGTAG |
| DHFR_ F | TCTGCGGCCGCCGCCAGGCTGTAAATCCCG |
| DHFR_R | ATCGCGGCCGCTCCTGCAAGTGCATAGAAGG |
| TGME49_245450_F | AATTCGGATCCATGCTGAACTTCATCCCGAAAAGATGCC |
| TGME49_245450_R | GAATTTTCTAGACTTGATGTTTTTCCCCTGGATGGGGTACG |
| TGME49_282180_F | AATTCGGATCCATGTCGCCGGTCGGACGCCTCTTTTTG |
| TGME49_282180_R | GAATTTGCTAGCTTTCGTCGTCGGGATGAAGACATCCGTG |
| TGME49_290030_F | AATTCGGATCCATGGGGCTCTCCCCGGCCTTC |
| TGME49_290030_R | GAATTTTCTAGAATGGTGTCCAGCGGCTTCCTC |
| TGME49_223040_F | AATTCGGATCCATGGCAGAGACTCGCGAAGGG |
| TGME49_223040_R | GAATTTTCTAGAAGAGTACTGCAGATCGGGCGCTCC |
